# Quantification of In Vitro Replicative Lifespan Elongation Activity of Hormones, Antioxidants, Plant Extract and Bacterial Exudate by Updated “Overlay Method”

**DOI:** 10.3390/medicines13020012

**Published:** 2026-03-30

**Authors:** Hiroshi Sakagami, Masayo Abe, Megumi Inomata, Hideki Aoyagi, Takao Tsukahara, Kenjiro Bandow, Shogo Nishino, Hiroshi Kadokura, Yuka Kato, Satoshi Yokose

**Affiliations:** 1 Meikai University Research Institute of Odontology (M-RIO), Meikai University School of Dentistry, Sakado 350-0283, Saitama, Japan; 2 Division of Microbiology, Meikai University School of Dentistry, Sakado 350-0283, Saitama, Japan; abe-0306@dent.meikai.ac.jp (M.A.); inomata@dent.meikai.ac.jp (M.I.); 3 Microbiology Research Center for Sustainability (MiCS), University of Tsukuba, Tsukuba 305-8572, Ibaraki, Japan; aoyagi.hideki.ge@u.tsukuba.ac.jp; 4 Division of Biochemistry, Meikai University School of Dentistry, Sakado 350-0283, Saitama, Japan; tsukahara@dent.meikai.ac.jp (T.T.); kbando@dent.meikai.ac.jp (K.B.); 5 Division of Fundamental Physics, Meikai University School of Dentistry, Sakado 350-0283, Saitama, Japan; s.nishino@dent.meikai.ac.jp; 6 Division of Endodontics and Operative Dentistry, Meikai University School of Dentistry, Sakado 350-0283, Saitama, Japan; h-kadokura@dent.meikai.ac.jp (H.K.); yuka-kato@dent.meikai.ac.jp (Y.K.); s-yokose@dent.meikai.ac.jp (S.Y.)

**Keywords:** anti-aging, replicative lifespan, overlay quantification method, hormones, antioxidants, hormesis

## Abstract

**Background/Objectives**: Many products that claim to have anti-aging effects have been reported, but their relative potency is not clear. In this study, the in vitro replicative lifespan extension (RLE) activity of various groups of physiologically active substances was compared by using the updated “overlay method”. **Methods**: Human dermal and periodontal ligament fibroblasts (HDFa, HPLF) were inoculated into the inner 60 wells of 96-well microplate, surround by sterile water to prevent the water evaporation. At Day 1 and Day 8, the cells were overlayed with wide ranges of concentrations (0.01–100 µM) of samples without medium change. Viable cell number was measured by the MTT method at Day 15 and then corrected for the variation in cell growth due to the location of inoculated cells. The RLE value was calculated as the maximum cell proliferation rate relative to the control. **Results**: Cell density of HDFa and HPLFs at subculture decreased with the passage number, and their growth was stopped at 56 or 85 population doubling levels (PDLs), respectively. Hydrocortisone showed the highest RLE values among six hormones, followed by three plant extracts, sodium ascorbate and quercetin. On the other hand, other antioxidants, chlorogenic acid, phenylpropanoids, vanilloids, and bacterial products showed little or no RLE effects. However, for HPLF cells, hydrocortisone did not show RLE effects while oxytocin showed slight stimulation. **Conclusions**: When differences in proliferation due to cell seeding position were corrected, the biphasic dose response curve of most of the compounds significantly reduced. The present study suggests the significant role of hormones for the regulation of the long-term aging process. To confirm systemic or clinical anti-aging effects, further in vitro and in vivo experiments are needed.

## 1. Introduction

**Figure 1:**
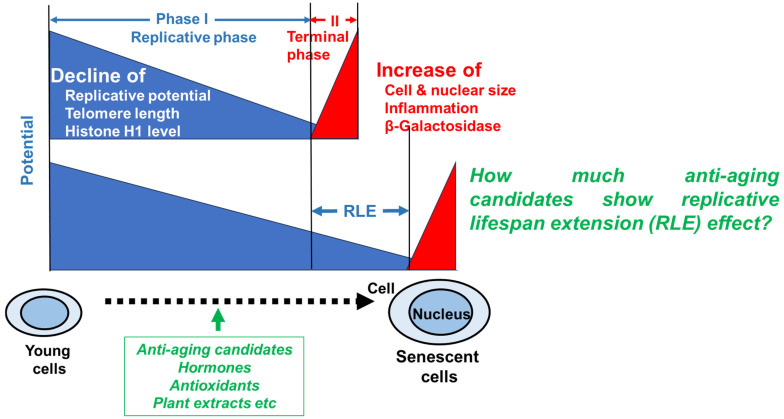
Expression of age-related characteristics during the in vitro aging process of human fibroblast model.

Due to the increase in lifespans, the proportion of elderly people has increased. As aging progresses, oxidation, inflammation, and cellular dysfunction occur, leading to the onset of various diseases and death [1,2]. Traditional medicines, natural products and even daily supplements may prevent age-related diseases, but their relative anti-aging potential has never been investigated. Human primary cultured cells are used as a model of cellular aging because they cease proliferation after approximately 50 ± 10 divisions due to DNA damage and deviation from the cell cycle. During the replicative phase (phase I), cellular metabolic activity, intracellular NAD^+^ concentration, telomere length and the extracellular matrix (e.g., collagen and elastin) gradually decline, while cellular and nuclear volume increase [3,4] (Figure 1). At the terminal phase (phase II), senescent cells exhibit a senescence-associated secretory phenotype (SASP) and affect the surrounding environment by secreting various physiologically active substances and inducing inflammation and tissue remodeling [5]. Associated β-galactosidase (SA-βgal) has been used as a marker of the terminal phase of aging, since this marker is expressed by larger senescent cells that occupy a very small percentage of the total population, but not smaller pre-senescent fibroblasts [6,7]. We found that β-galactosidase can be detected only in dying or irreversibly damaged cells, which cannot be evaluated by RLE (Figure 1). The phase I process is overwhelmingly longer than the phase II process, and we thought that the length of phase I may reflect “healthy life expectancy”, which refers to the period during which one can live independently and healthily without restrictions on daily life. Additionally, we got the idea that establishing a method to accurately measure this replicative lifespan extension (RLE) could be used to search for substances that extend healthy lifespan. Although in vitro RLE is a valid evaluation item for “anti-aging”, it only captures one aspect of cellular aging, primarily proliferation ability, and the connection of RLE and the expression of other aging markers should be further consulted.

Until now, the colony formation method has been used to quantify the extension of the lifespan of human fibroblasts, based on the tight association of aging progression and reduction in colony forming activity. However, there are colonies of various morphological types (dense, mixed and diffuse) formed by skin fibroblasts. During in vitro aging, diffuse colonies, which contain a higher number of SA-βgal-positive senescent cells, and a lower number of rapidly grown Ki67-positive cells, increase [8]. This heterogeneity of colony size hinders the accurate measurement of RLE activity. This urged us to establish a new quantitative method of the RLE activity using 96-well microplates [9]. After the publication, however, we noticed that two additional restrictive conditions have to be added for the accurate quantification of the RLE: first, the number of inoculated cells have to be within a quantitative range at Day 15, and second, the variation in cell proliferation rate due to the position of the inoculated cells must be corrected.

In this study, we first updated the RLE quantification method as described above, then identified hydrocortisone as the positive control that showed the highest RLE activity for human dermal fibroblasts (HDFa). Next, using hydrocortisone as the positive control, the RLE activity of various physiologically active compounds including antioxidants with anti-aging properties was investigated. We also examined their effect on periodontal ligament fibroblasts (HPLFs) for their future dental application.

## 2. Materials and Methods

### 2.1. Chemicals

Hydrocortisone (MW = 362.5), estradiol (MW = 272.4), resveratrol (MW = 228.1), curcumin (MW = 368.4), quercetin (MW = 302.2), sodium ascorbate (MW = 198.1), vanillic acid (MW = 168.1), vanillin (MW = 152.2), *p*-coumaric acid, caffeic acid (MW = 180.1), trans-ferulic acid (MW = 194.2), and chlorogenic acid (MW = 354.3) were purchased from Tokyo Chemical Industry Co., Ltd., Tokyo, Japan. hydrocortisone sodium succinate (Solu-Cortef) (MW = 485.5) was purchased from Takeda Pharmaceutical Co. Ltd., Osaka, Japan; fetal bovine serum (FBS) and 3-(4,5-dimethylthiazol-2-yl)-2,5-diphenyltetrazolium bromide (MTT) were purchased from Sigma-Aldrich Inc., St. Louis, MO, USA. Dimethyl sulfoxide, modified Eagle’s medium (DMEM), penicillin–streptomycin solution (×100), and 0.25% trypsin-1 mM EDTA-4Na, testosterone, melatonin, and oxytocin were purchased from Fujifilm Wako Pure Chemical Ind., Osaka, Japan, while 96-microwell plates were purchased from Techno Plastic Products AG, Trasadingen, Switzerland [9].

### 2.2. Preparation of Plant Extracts

Taheebo tea was purchased from Konishi Pharmaceutical Co., Ltd. (Osaka, Japan). First, 10 g of tea was weighed out and placed in a tea bag in a heat-resistant pot containing 1 L of water. The tea was brought to a boil, then reduced to low heat and brewed for 30 min. After making the extract isotonic by adding phosphate buffered solution (PBS(-)powder, it was sterilized by filtration through a 0.45 µm membrane filter. Twenty Japanese pine cones of *Pinus parviflora* Sieb et Zucc were extracted with 4 L boiling water, and made isotonic, and then sterilized similarly. The alkaline extract of *Sasa* sp. (Sasa Health^®^) (SE) was provided from Daiwa Biological Research Institute Co., Ltd., Kanagawa, Japan.

### 2.3. Preparation of Bacterial Cells and Supernatant

#### 2.3.1. Intestinal Bacteria

Microorganisms at late logarithmic growth phase or early stationary phase were used. *Akkermansia muciniphila* (grown in modified GAM + glucose + acetate), *Bifidobacterium longum* spp. *longum* and *Lactobacillus paragasseri* (both grown in GAM broth) were grown under anaerobic conditions. *Lactococcus lactis* spp. *cremoris* (grown in MRS broth) was grown under aerobic conditions. The culture medium was centrifuged (6000× *g*, 5 min) to collect the bacterial pellet. The collected bacterial cells were frozen and stored at −80 °C. The freeze-dried product was crushed and extracted with either pure water or 75% (*v*/*v*) ethanol (Table 1), according to previous reports [10,11]. Only *L. lactis* spp. *cremoris* was treated with gold nanoparticles after removing the medium components by centrifugation. Freeze-dried feces (intestinal bacterial community) of Japanese bush warbler (Uguisu-no-kona) was directly extracted with 75% ethanol. All samples were filtered through a Millipore filter (0.22 μm) for sterilization.

#### 2.3.2. Oral Bacteria

*Candida albicans* strain SC5314 (MYA-2876) was cultured in YEPD broth under aerobic conditions at 37 °C. *Porphyromonas gingivalis* (ATCC 33277) was cultured anaerobically in GAM broth supplemented with 0.5 mg/mL hemin and 5 mg/mL menadione using AnaeroPack systems. The supernatants were collected and centrifuged again at 15,000 rpm for 30 min at 4 °C. The resulting supernatants were filtered (0.22 μm) and ultracentrifuged at 100,000× *g*.

### 2.4. Cell Culture

**Figure 2:**
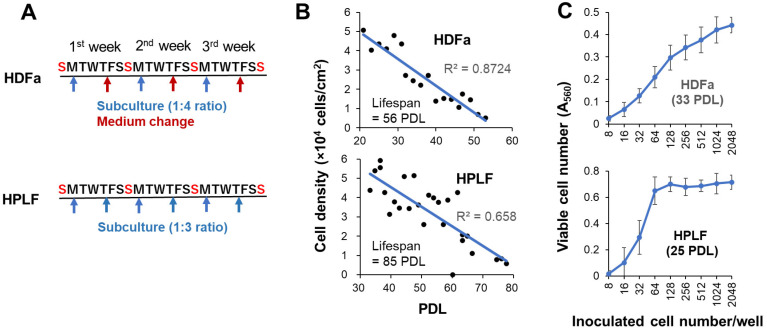
Subculture schedule of HDFa and HPLF cells (**A**) to monitor the aging-associated decline of viable cell number (**B**) and the range of linearity of viable cell number at Day 15 (**C**).

Human dermal fibroblasts (HDFa) from adult skin (catalog number: C0135C; Thermo Fisher Scientific, Waltham, MA, USA) and human periodontal ligament fibroblasts (HPLFs) (purchased from SCR ScienCell Research Laboratories, Carlsbad, CA, USA) were cultured at 37 °C in regular culture medium [DMEM supplemented with 10% heat (56 °C, 30 min) inactivated FBS, 100 U/mL penicillin G, and 100 μg/mL streptomycin sulfate] in a humidified incubator (MCO-170 AICUVD-P; Panasonic Healthcare Co., Ltd., Gunma, Japan) with 5% CO_2_ [9]. For subculture, HDFa cells were harvested using 0.25% trypsin–EDTA and seeded at a 1:4 ratio once a week, with a medium change after 4 days. On the other hand, HPLF cells were seeded at a 1:3 ratio twice a week, considering rapid cell growth (Figure 2A). With continued passage in this manner, the cell density gradually decreased, and proliferation ceased after 56 or 85 divisions (Figure 2B).

### 2.5. Quantification of Replicative Lifespan Elongation (RLE) Activity

**Figure 3:**
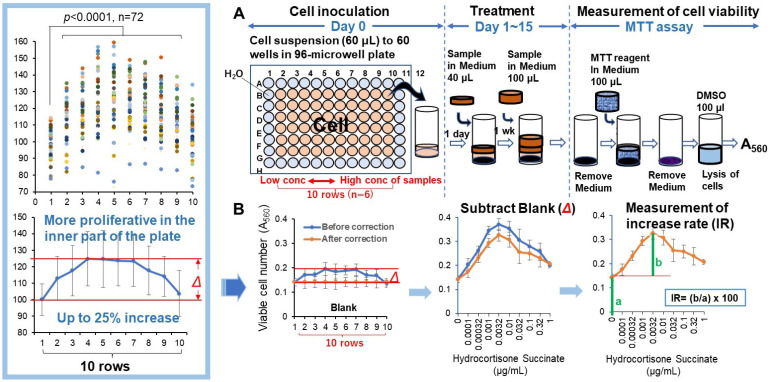
The determination of replicative lifespan elongation (RLE) by updated “overlay method”. Cells were inoculated into the inner 60 wells of a 96-microwell plate, and the test samples were treated for 14 days without (control) or with increasing concentrations of samples from the left to right (*n* = 6), with the sample layer added during the incubation. After culturing for two weeks, the viable cell count was measured (**A**). Since cells grow more vigorously closer to the center of the plate, the variation in cell number due to location was corrected0 in each experiment (**B**) and insert. The symbols in various colors represent the 72 data points arranged in a random order. Δ indicates the difference from the outermost value. a represents the A_560_ value of the control (blank) cells after 14 days. b indicates the increase in A_560_ at the concentration where the cell number reaches its maximum.

Replicative lifespan elongation (RLE) activity was quantified by the updated “overlay” method. Before seeding the cells, 275 μL of sterile water was added to the outer periphery of the 96-microwell plate (Becton Dickinson Labware, Franklin Lakes, NJ, USA) to minimize water evaporation and bacterial infection during cell culture. Thus, 60 µL of 32–256 cells (depending on the PDL and type of cell) were inoculated onto the inner 60 wells of the plate. After 24 h, complete cell attachment was achieved, and 40 μL of fresh medium containing different concentrations of sample was added, without medium change to prevent cell detachment. After incubation for 7 days, cells were again overlaid with 100 µL of fresh medium with samples. Cells were incubated for a further 7 days (for a total of 14 days). The relative viable cell number at Day 15 was then determined by the MTT method and expressed as the absorbance at 560 nm (A_560_) [9] (Figure 3A). The A_560_ value of the control cells was usually in the range of 0.15 to 0.45 to keep the linearity of viability (Figure 2C). Since cells near the center showed more vigorous cell proliferation than the cells in the periphery (Figure 3B, insert), the difference in growth rate (Δ) (up to 25%) depending on the location of cell inoculation was corrected. The replicative lifespan elongation (RLE) was calculated by the following equation: RLE = (b/a) × 100, where a is the A_560_ of control cells, and b is that of the maximum increase in the cell number of treated cells at the optimal concentrations of the sample (Figure 3B).

### 2.6. Statistical Analysis

Experimental data are expressed as the mean ± standard deviation of sextuple determinations. Since we compared the means of the control group with those of the other groups, Dunnett’s post hoc analysis, rather than Bonferroni and Tukey analyses, was performed after one-way ANOVA to compare the means of 10 samples including the control, using the SPSS program for Windows version 22, and *p* < 0.05 was considered to be significant. Using the Shapiro–Wilk test, we first confirmed that each group obeyed the normal distribution. Since the variances of each group had different values, the Welch-type Dunnett’s test or the heteroscedastic Dunnet’s test was used instead of the usual Dunnet’s test.

## 3. Results

### 3.1. Requirement for Accurate Calculation of Replicative Lifespan Extension (RLE) Activity

#### 3.1.1. Inoculation of Optimal Concentration of Cells to Allow Qualitative RLE Measurement

After long-term culture (2 weeks), it is necessary to bring the cell number within a range that permits quantitative measurement. For HDFa cells, the A_560_ value at Day 15 should be between 0.1 and 0.42, which corresponds to 16 to 1024 cells/well (Figure 2C, upper panel; Supplementary Figure S1).

Also, for HPLF cells, linearity was kept similarly between A_560_ values of 0.1 and 0.6, but the optimal inoculation number shifted to a lower range (16 to 64 PDL) due to their rapid growth when using young cells (25 PDL, 40% lifespan) as compared with HDFa cells. If an excess of cells is inoculated, the number of cells will fall outside the range of quantitative measurement, making it impossible to accurately determine the cell number (Figure 2C, lower panel). When older HPLFs are used, more cells should be inoculated to fall into the linear range.

#### 3.1.2. Correction for Proliferation Errors Due to the Position of Cell Inoculation Is Essential

We found that the viable cell number increased gradually when the position of cell inoculation approached the center. The difference in viable cell number between these two points was approximately 25% on the average of 72 experiments (Figure 3B). This artifactual variation should be corrected in each well. If this correction was not performed, all cells would exhibit a biphasic hormesis curve, even without the addition of test samples, leading to the misconception that the sample promotes proliferation.

### 3.2. RLE Activity of Anti-Aging Candidates Against HDFa Cells

#### 3.2.1. Hormones

**Figure 4:**
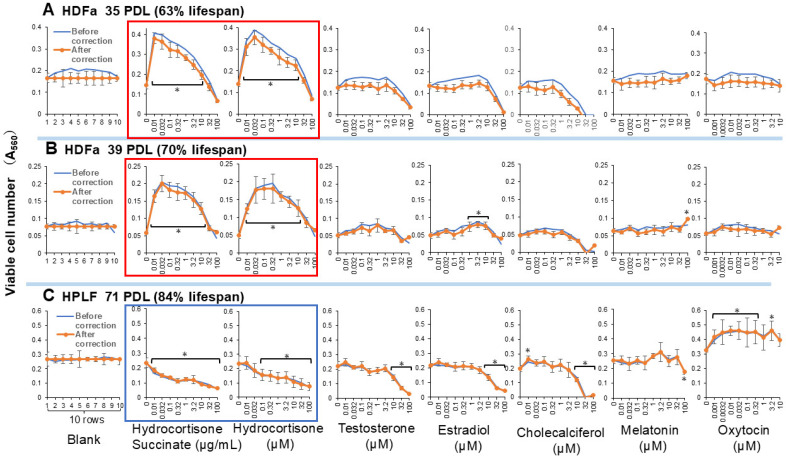
RLE activity of 6 hormones against human dermal (HDFa) (**A**,**B**) and periodontal ligament fibroblasts (HPLF) (**C**). Each point represents mean ± S.D. (*n* = 6). * *p* < 0.05 vs. control (Dunnett’s post-test). RLE values are presented in Table 1. The effects of hydrocortisone can be reversed depending on the cell type, as illustrated by the red and blue boxes.

Among six hormones, both water-soluble and lipophilic hydrocortisone, but not succinate (Supplementary Figure S2), exhibited the highest replicative lifespan extension (RLE) effects on human dermal fibroblast (HDFa) cells. The RLE effects of other hormones (testosterone, estradiol, cholecalciferol, melatonin, and oxytocin) were negligible (Figure 4, Supplementary Table S1). Hydrocortisone, either hydrophilic or lipophilic, can be used as a positive control for the search of new RLE substances for HDFa cells. When the treatment time was halved to 7 days, the RLE effect of hydrocortisone was also halved (Supplementary Figure S2), indicating that the RLE effect of hydrocortisone increases continuously for at least 2 weeks.

Interestingly, hydrocortisone did not increase, but rather reduced the replicative lifespan of human periodontal ligament fibroblasts (HPLFs). On the other hand, oxytocin exhibited a slight RLE effect on HPLFs (Figure 4C, Supplementary Table S1).

#### 3.2.2. Antioxidants

Among eight antioxidants, only sodium ascorbate and quercetin showed some RLE effects, but they were not as potent as hydrocortisone. Other antioxidants such as curcumin, resveratrol, astaxanthin, docosahexaenoic acid (DHA), coenzyme Q10 and epigallocatechin gallate (EGCG) were essentially inactive (Supplementary Figure S3, Supplementary Table S1).

#### 3.2.3. Chlorogenic Acid, Phenylpropanoids and Vanilloids

**Figure 5:**
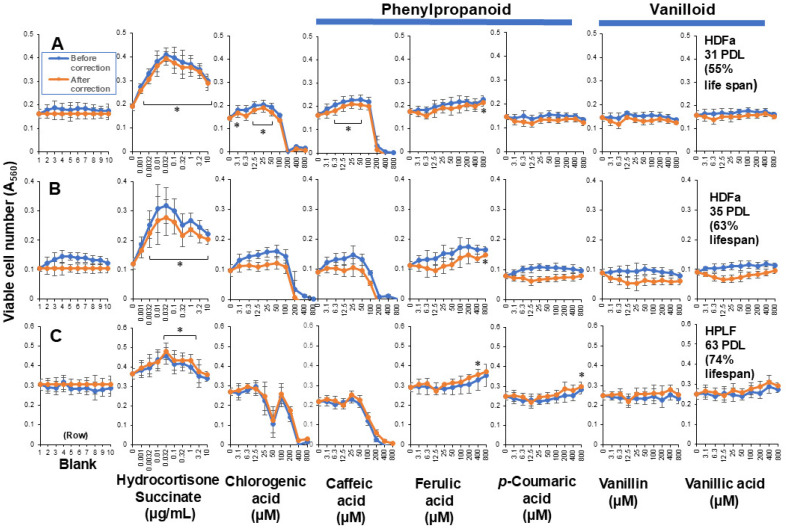
RLE activity of phenylpropanoids and vanilloids on HDFas (**A**,**B**) and HPLFs (**C**). Each point represents mean ± S.D. (*n* = 6). * *p* < 0.05 vs. control (Dunnett’s post-test).

Since we reported that coffee extract showed very high RLE activity against HDFa cells [9], we investigated the RLE activity of the main component of coffee. In contrast to our expectation, chlorogenic acid, caffeic acid, and other related phenylpropanoids (ferulic acid and *p*-coumaric acid), and vanilloids (vanillin and vanillic acid) showed little or no RLE effects on HDFa cells (Figure 5, Supplementary Table S1).

#### 3.2.4. Plant Extract

Taheebo tea showed comparable RLE activity with hydrocortisone in HDFa cells, but the effective concentration range was slightly narrower. Pine cones of *Pinus parviflora* Sieb. et Zucc. and *Sasa* sp. extracts showed comparable RLE values (Supplementary Figure S4A,B). Only *Sasa* sp. showed weak RLE activity of HPLFs (Figure S4C, Table 1). It remained to fractionate the plant and purify the active components to finally conclude whether it is due to the synergistic effect of plant components or a single component.

#### 3.2.5. Bacterial Secretion

**Figure 6:**
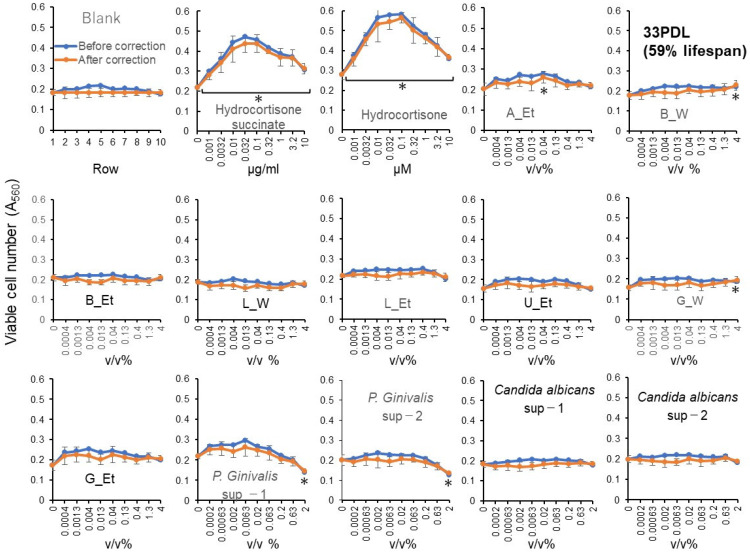
RLE activity of bacterial culture supernatants. Each point represents mean ± S.D. (*n* = 6). * *p* < 0.05 vs. control (Dunnett’s post-test). RLE values are presented in Table 1.

Six of the eight intestinal bacteria, and one out of four oral bacteria showed minor RLE activity, although their effects are much lower than hydrocortisone (Figure 6, Supplementary Table S1).

## 4. Discussion

Aging is driven by various hallmarks such as genomic instability, epigenetic changes, telomere attrition, loss of protein homeostasis, mitochondrial dysfunction, cellular senescence, stem cell depletion, chronic inflammation, and dysbiosis [12]. Without a positive control identified, it is impossible to culture many candidate substances simultaneously for as long as one to two months—until the cells stop proliferating—in order to search for new anti-aging compounds. The purpose of this paper is to establish a method to quantitatively measure the replicative lifespan extension (RLE) activity for a relatively short period of time (2 weeks). Then, the RLE values of various anti-aging candidates against human dermal fibroblast were investigated to explore new skincare substances.

During the establishment of the updated assay for RLE activity, we had noticed that the following four conditions must be met for accurate calculation of RLE values: (i) preparation of single-cell suspension to prevent the peeling of the aggregated cells during long culture and minimize the variation in the data as much as possible, (ii) inoculation of an appropriate number of cells to allow the quantitative measurement of viable cell number, (iii) overlay of fresh medium containing samples to prevent nutritional depletion and cell detachment by the medium change, and (iv) the correction of different levels of cell growth in the different wells of the 96-microwell plate (Figure 3).

**Figure 7:**
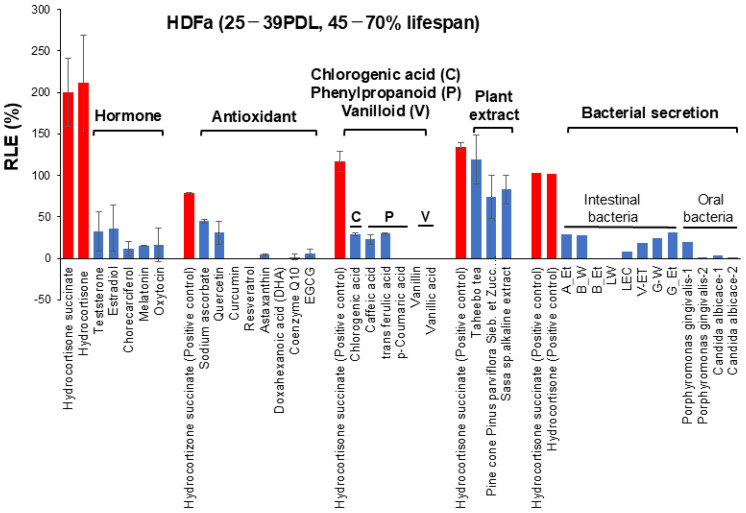
Relative potency of RLE activities of test samples on HDFa and HPLF. These data were derived from Figure 4, Figure 5 and Figure 6, Supplemental Figures S3 and S4, and Table S1. Exact values (except for bacterial sections) are the mean of two independent experiments (each performed with 6 technical replicates). Data of antioxidants and plant extract were derived from Supplementary Figure S3 and Figure S4, respectively. Red bar indicates positive control (i.e., hydrocortisone succinate or hydrocortisone). Blue bar indicates test samples.

The present study, performed under these four restricted conditions, demonstrated that hydrocortisone showed the highest RLE values among six hormones against HDFa cells, followed by several plant extract such as taheebo tea, pine cone and *Sasa* sp., whereas most antioxidants, chlorogenic acid, phenylpropanoids, vanilloid and bacterial culture supernatants showed little or no RLE effects. These data are summarized in Figure 7 and Supplementary Table S1. Many drugs are known to exhibit a biphasic dose–response curve, so-called hormesis, over a wide concentration range [13]. However, such a biphasic dose response of antioxidants was significantly diminished, if the artificial effect of increased cell growth in the center of the plates was subtracted. The reproducibility of the present findings should be investigated in long-term culture experiments with various types of cells.

Initially, we examined the RLE activity of various physiological chemicals using human dermal fibroblast (HDFa) cells. Dermal fibroblasts are the main cell type present in skin connective tissue (dermis), and play an important role in cutaneous wound healing [14]. Hydrocortisone, the endogenous glucocorticoids in human, is released through the hypothalamic–pituitary–adrenal axis in response to various stressors. The in vitro anti-aging effect of hydrocortisone changes depending on the type of vertebrate cell lines. It has been reported to prolong the lifespan of human lung fibroblasts by the reduction in its high-affinity binding sites [15]; additionally, it accelerates skin aging [16] (PMID: 28793178), causes skin atrophy and inhibits collagen production [17]. We also found that hydrocortisone did not clearly show RLE activity in human periodontal ligament fibroblasts (HPLFs) (Figure 4C and Figure 5C). HPLFs are fibroblasts specialized in immune and inflammatory control in periodontal tissues. As far as we know, no studies were found that directly compared the toxicity of glucocorticoids to HDFa cells and periodontal ligament fibroblasts (PDLFs). It has been reported that glucocorticoid caused the translocation of its receptor into the nucleus and induced dickkopf-1 (DKK-1) and thereby deteriorated periodontal ligament stem cells [18]. Therefore, it is highly likely that differences in the effectiveness (sensitivity) of steroid-induced inflammation suppression may arise through differences in the expression levels, isoforms, and coactivators of glucocorticoid receptors, as well as differences in baseline activity and crosstalk of the NF-κB/MAPK pathway. These findings suggest that glucocorticoids may affect the proliferation, differentiation, and immune response of periodontal ligament cells, thereby disrupting periodontal tissue homeostasis [18].

The glucocorticoid receptor (GR) is present in the cytoplasm due to an anchoring complex that has a high affinity for glucocorticoids. Glucocorticoids cross the membrane where they can be activated/inactivated by 11β-hydroxysteroid dehydrogenase (11β-HSD) isoforms. The binding of active glucocorticoids to a glucocorticoid receptor (GR) leads to dissociation of the complex. GRs can show nongenomic effects in the cytoplasm, but mostly enter the nucleus. The GR binds to chromatin to directly activate or repress transcription by cofactors, genes, or other transcription factors. Glucocorticoids act on almost all cells, but responses vary significantly between cell types due to factors such as transcription factor networks, chromatin state [19], and changes in GRα/GRβ isoforms [20].

On the other hand, oxytocin showed a weak but significant RLE effect on HPLFs (Figure 4C). Oxytocin is strongly expressed in oral cells, such as periodontal ligament fibroblasts [21] and pulp cells [22], suggesting a connection between RLE activity and receptor expression. It has been reported that oxidation products accumulate with age, and that oxidative stress accelerates aging [1,2]. However, this study provided a surprising result that antioxidants showed very low or no RLE activity. A short period (1 day) of treatment of human dermal papilla cells with higher concentrations (50–100 µM) of resveratrol has been reported to downregulate the expression of aging markers such as β-galactosidase and the senescence-associated secretory phenotype (SASP) [23]. On the other hand, a longer (2 weeks or more) treatment period with lower concentrations (0.2–20 µM) of resveratrol showed a weak RLE effect on human fetal lung fibroblast MRC-5 (two PDLs) and no effect on human foreskin fibroblasts FB0603, but rather shortened the lifespan of human ear skin fibroblasts H8F2p 25 LM and human foreskin fibroblasts Hs68 [24]. A recent study demonstrated that nitroxide 4-hydroxy-TEMPO, ergothioneine, and Trolox extended the replicative lifespan (40 ± 1 population doublings (PDs)) by 7 ± 2, 4 ± 1, and 3 ± 1 PDs, suggesting the possible involvement of the elevation of the mitochondrial membrane potential in the prolongation of the replicative lifespan, whereas coumaric acid, curcumin and resveratrol did not affect the replicative lifespan [25]. These data are consistent with the present study that showed no significant RLE activity of resveratrol on HDFa cells.

**Figure 8:**
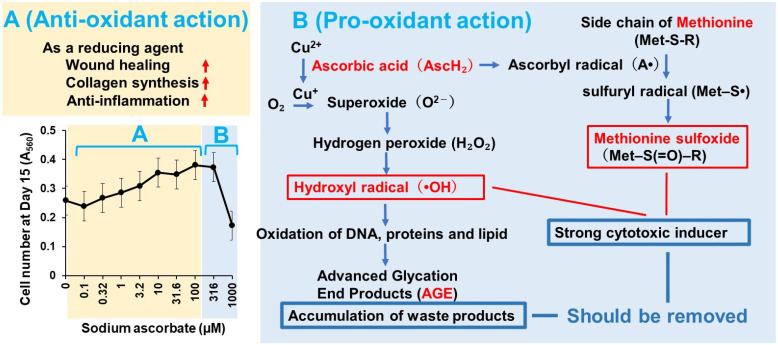
Biphasic action of sodium ascorbate. Anti-oxidant actions were indicated by red arrows. Pro-oxidant actions were indicated by blue boxes.

One possible reason is that antioxidants and polyphenols are unstable under long culture conditions, possibly due to their decomposition and/or production of various oxidized products in the presence of oxygen [26,27]. Ascorbic acid (vitamin C) generates hydrogen peroxide and methionine sulfoxide [28]. The production of these stress markers rises dramatically with increasing concentrations of antioxidants. Thus, ascorbic acid shows biphasic actions, showing beneficial effects (such as would healing, collagen synthesis and anti-inflammation) at lower concentrations, while it has harmful effects (such as oxidation of macromolecules and accumulation of waste oxidation products) at higher concentrations (Figure 8). It is also necessary to check whether medium nutrients may be depleted during long-term culture. To address this issue, frequent administration of antioxidants and regular medium changes are recommended. Also, the addition of catalase (which degrades hydrogen peroxide) or other antioxidant enzymes (superoxide dismutases—SODs and catalase, glutathione peroxidase—GPx) [29] may ameliorate the harmful effect of oxidative stress. Citric acid contributes to the stabilization of vitamin C, so it is expected that the antioxidant power will be increased if the two compounds are used together at low concentrations that do not cause toxicity [30].

We have previously reported that lignin degradation products such as phenylpropanoids (caffeic acid, ferulic acid and *p*-coumaric acid) and especially vanilloids (vanillic acid and vanillin) showed higher anti-UVC activity (possibly due to the hydroxyl radical scavenging activity) than vitamin C, due to higher stability than sodium ascorbate [31]. This suggests that substances with high anti-UVC activity (short effect) do not always show RLE activity (long effect).

Several plant extracts such as taheebo tea, pine cone and *Sasa* sp. show higher RLE effects than their components. The combination of several components may potentiate their RLE effect. In dentistry, there are many hot water-extracted herbal preparations (so-called Kampo Medicines), such as Rikkosan, used for the treatment of toothache [32], and Hangeshashinto, for the treatment of stomatitis [33]. Sakagami et al. demonstrated that Rikkosan exhibited RLE effects on HDFa cells, comparable with those achieved by hydrocortisone, but its optimal concentration range is narrow. On the other hand, Hangeshashinto did not exhibit RLE activity, due to the cytotoxic effects of constituent plant extracts. The removal of cytotoxic substances may elevate the specific activity of these two Kampo Medicines (The 67th Annual Meeting of Japanese Association for Oral Biology, September 2025). We found that several intestinal bacteria showed slightly higher RLE activity on HDFa cells than oral bacteria. Their effects on human oral cells such as gingival fibroblast and pulp cells, as well as HPLFs, are under way.

The MTT assay primarily measures mitochondrial enzyme activities such as succinate dehydrogenase and NAD(P)H-dependent dehydrogenase [34]. MTT is taken up into cells and reduced to purple formazan by mitochondrial dehydrogenases. Because succinate dehydrogenase is strongly involved, the MTT assay can be understood as an indirect measure of cell viability. However, MTT cannot measure the effects of various factors that affect lifespan (such as chronic inflammation, DNA damage, and the accumulation of senescent cells). Furthermore, transiently elevated metabolism does not directly correlate with long-term health or lifespan. Thus, we extended the incubation time up to 2 weeks, comparable to the colony formation assay. We found that hydrocortisone is likely to extend the replicative lifespan of HDFa cells. Experiments are now needed to determine whether this is due to long-term cell proliferation, i.e., reversing the senescence-associated decrease in cell density or maintaining the mitochondrial enzyme activity over time.

Although antioxidants have been reported to play roles such as redox buffering, modulation of signal transduction, and prevention of cellular damage, these effects are observed only over short time spans. In the long-term processes involved in aging, it is unlikely that these actions can be sustained. In addition, changes in factors such as tissue type, sex, age, and environment may alter the effects of antioxidants themselves. To conclude that most antioxidants exhibit little or no RLE activity, it is necessary to demonstrate that repeated administration of the antioxidant does not improve the condition. Repeated overlaying of the culture medium places a significant gravitational load on the cells, which may affect their normal metabolic capacity. Also, the cell detachment caused by repeated medium changes must be considered.

## 5. Conclusions

We have developed the updated “overlay method” that can measure the RLE activity of a large number of samples at the same time. The determination of RLE values by the MTT method is based on mitochondrial enzyme activities. Since there is a close relationship between mitochondrial dysfunction and senescence [35], this system may be useful for developing drugs that extend the healthy lifespan. In this study, hydrocortisone exerted an RLE effect in dermal fibroblast, but inhibited it in periodontal ligament cells, suggesting the existence of a tissue-specific regulatory mechanism. In contrast, the low RLE values of many antioxidants may be due to the short-term nature of their effects, suggesting the importance of establishing a strategy of how to stabilize them. Furthermore, because various plant extracts contain a large number of active ingredients, a strong synergistic effect may be observed. Conversely, if cytotoxic substances are present, removing them may enhance their activity. The present method can be useful for the purification and identification step of new substances that extend replicative lifespan. Aging in the body is a complex phenomenon involving not only multiple cells, multiple organs, immune systems, metabolism, and hormones, but also cell–cell interactions, inflammation, blood flow, and the nervous system. Therefore, cellular effects do not necessarily translate directly into systemic or clinical anti-aging effects. To reach clinical trials, further in vitro studies are necessary to investigate the expression of various aging markers (such as p16, SA-β-gal, and DNA damage), the signaling pathways, and to perform animal experiments to check the safety and drug metabolism of candidate substances with the highest RLE activity.

Using the updated “overlay method”, we systematically compared the replicative lifespan effects of different substance classes (hormones, antioxidants, plant extracts, and bacterial exudates) and summarized them in Table 2. The present approach can also be applied to other types of primary cultured cells—such as epithelial cells, endothelial cells, and stem cells—as long as they have the ability to adhere to plates and proliferate under optimal culture conditions. With larger plates, it may become difficult to perform statistical analysis because many samples cannot be measured simultaneously. In addition, when working with organoids, preliminary experiments are required to establish a method for equalizing the number of cells. The present application to dermal fibroblasts and periodontal ligament fibroblasts has practical implications for esthetic medicine and dentistry.

We are considering the following steps for our research: We will examine whether the effects of hydrocortisone differ between dermal and epidermal cells in the skin, and between the sexes of donors (obtained from a cell bank), and we will investigate the effects of glucocorticoid receptor antagonists. For cells from other tissues, we will first identify positive controls. For plant components, we plan to fractionate and purify the active ingredients and examine the possibility of synergistic effects between each component.

## Figures and Tables

**Table 1:** Culture and extraction conditions of bacteria used in the present study.

Sample	*Bacteria*	Medium	Extracted by
A_Et	*A. muciniphila*	GAM + glucose + acetate	75% EtOH
B_W	*B. longum* spp. *longum*	GAM broth	Hot water
B_Et	*B. longum* spp. *longum*	GAM broth	75% EtOH
L_W	*L. paragasseri*	GAM broth	Hot water
L_Et	*L. paragasseri*	GAM broth	75% EtOH
U_Et	Uguisu-no-kona (feces)	(-)	75% EtOH
G_W	*L. lactis* spp. *cremoris*	MRS broth	Hot water
G_Et	*L. lactis* spp. *cremoris*	MRS broth	75% EtOH

**Table 2:** RLE activity of four representative groups of substances.

		RLE Activity			
Group		High	Medium	Low	None
Hormone	Hydrocortisone	●			
	Testosterone		●		
	Estradiol		●		
	Cholecalciferol			●	
	Melatonin			●	
	Oxytocin			●	
Antioxidant	Sodium ascorbate		●		
	Quercetin			●	
	Curcumin				●
	Resveratrol				●
	Astaxanthin				●
	Doxahexanoic acid (DHA)				●
	Coenzyme Q10				●
	EGCG				●
	Chlorogenic acid			●	
(Phenylpropanoid)	Caffeic acid			●	
	Ferulic acid			●	
	*p*-Coumaric acid				●
(Vanilloid)	Vanillin				●
	Vanillic acid				●
Plant extract	Taheebo tea	●			
	Pine cone Pinus parviflora Sieb. et Zucc. extract	●			
	*Sasa* sp. alkaline extract	●			
(Kampo)	Rikkosan	●			
(Kampo)	Hangeshashinto				●
Bacterial exudate	Intestinal A_Et, B_W, V-ET, G-W, G_Et			●	
	Intestinal B_Et, LW, LEC				●
	*Porphyromonas gingivalis*-1			●	
	*Porphyromonas gingivalis*-2				●
	*Candida albicace*-1, *Candida albicace*-2				●

## Data Availability

The original contributions presented in this study are included in the article/Supplementary Material. Further inquiries can be directed to the corresponding author.

## Supplementary Materials

The following supporting information can be downloaded at: https://www.mdpi.com/article/10.3390/medicines13020012/s1, Figure S1: Linearity of RLE effect of hydrocortisone was maintained between 0.2 and 1.0 of A560 in HDFa cells; Figure S2: Hydrocortisone succinate, but not succinic acid, showed potent RLE effect on HDFa cells; Figure S3: Most antioxidants, except quercetin and sodium ascorbate, showed little or no RLE effect on HDFa cells; Figure S4: Taheebo, pine cone and Sasa sp. extracts showed potent RLE effects on HDFa cells; Table S1: RLE activities of hormones, antioxidants, polyphenols, and plant and bacterial exudates on HDFa and HPLF cells.

## Abbreviations

The following abbreviations are used in this manuscript:

RLE	Replicative lifespan elongation
PDL	Population doubling level
HDFa	Human dermal fibroblast
HPLF	Periodontal ligament fibroblast
GR	Glucocorticoid receptor
