# Supplementary material for: Quantification of In Vitro Replicative Lifespan Elongation Activity of Hormones, Antioxidants, Plant Extract and Bacterial Exudate by Updated “Overlay Method”
Source: Medicines (Basel). 2026 Mar 30;13(2):12. doi: 10.3390/medicines13020012 (PMC13108127; doi:10.3390/medicines13020012)
Supplement: Supplementary file 1 [file medicines-13-00012-s001.zip › medicines-4097392-supplementary.pptx]

## Slide 1
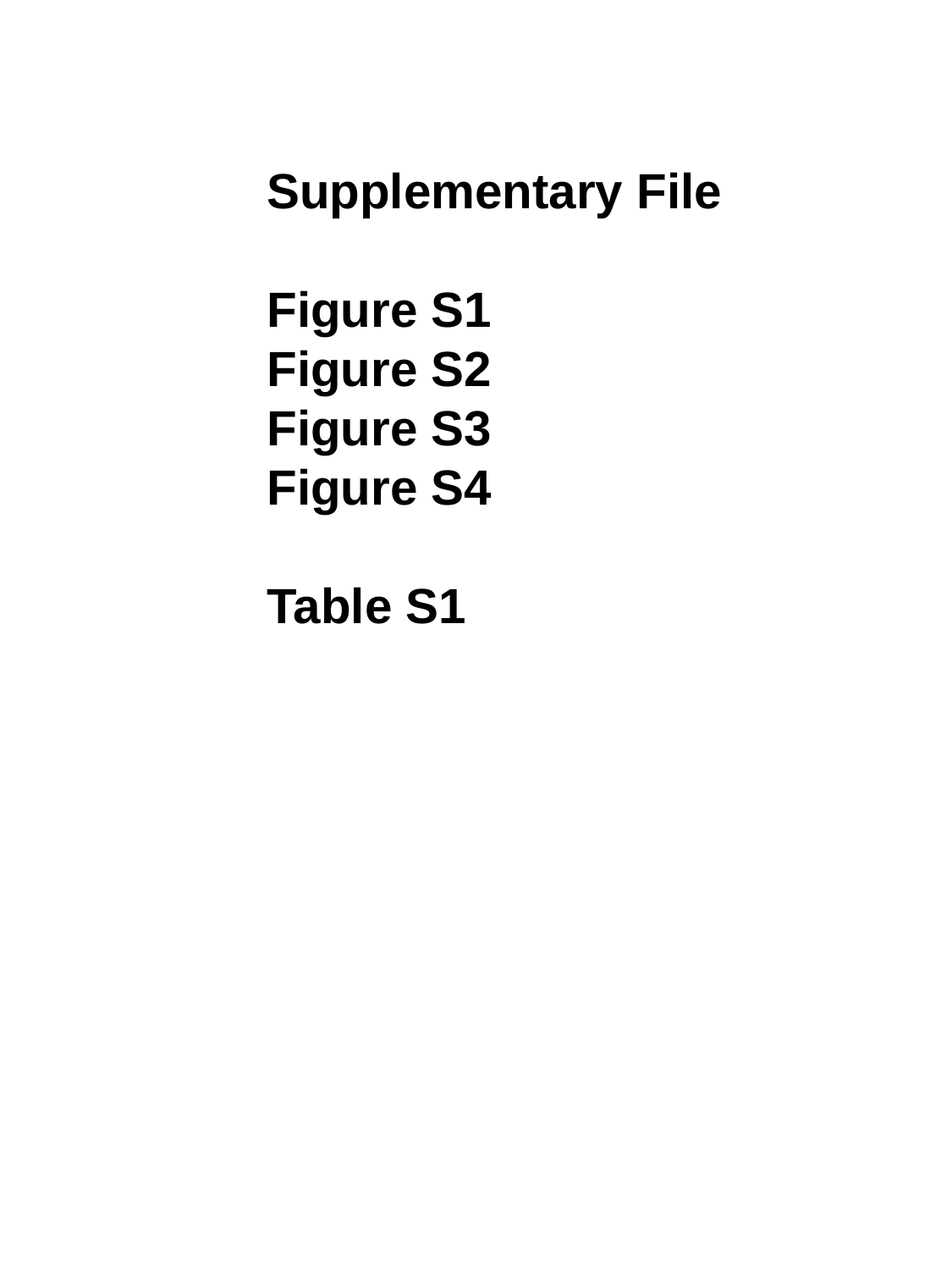

Supplementary File
Figure S1
Figure S2
Figure S3
Figure S4
Table S1

## Slide 2
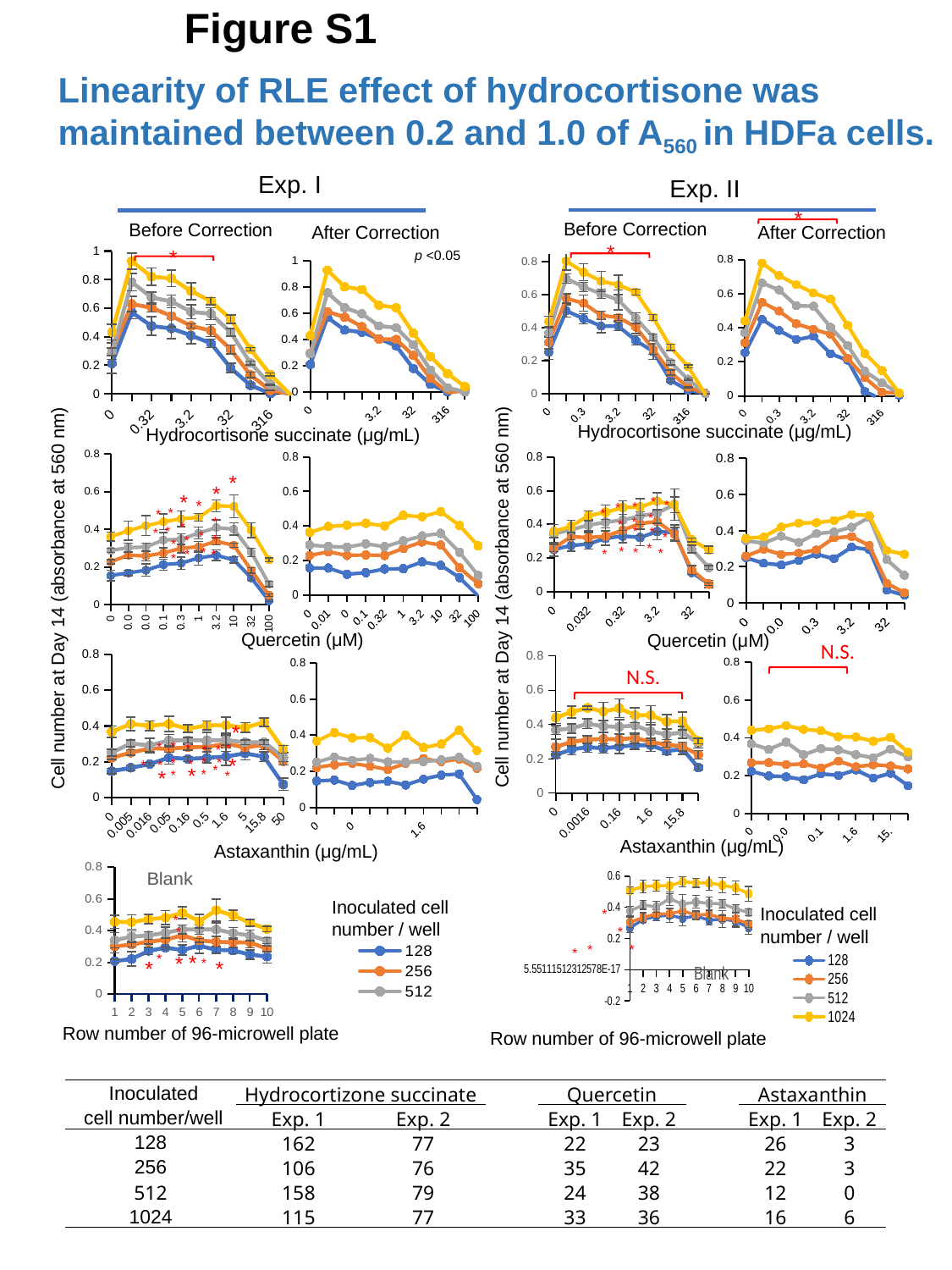

Figure S1
Linearity of RLE effect of hydrocortisone was maintained between 0.2 and 1.0 of A560 in HDFa cells.
Exp. I
Exp. II
Before Correction
Before Correction
After Correction
After Correction
### Chart
| Category | 128 | 256 | 512 | 1024 |
|---|---|---|---|---|
| 0 | 0.21225000048677126 | 0.2979666627943516 | 0.29428333913286525 | 0.43228333195050556 |
| 0.1 | 0.5707166753709316 | 0.6288999952375889 | 0.7819499907394251 | 0.9288333356380463 |
| 0.32 | 0.4746833307047685 | 0.6024833458165327 | 0.6744999922811985 | 0.8199833333492279 |
| 1 | 0.45784999057650566 | 0.5448499955236912 | 0.6464499967793623 | 0.8087166647116343 |
| 3.2 | 0.4088500055174033 | 0.47553333019216854 | 0.574333323786656 | 0.7194666663805643 |
| 10 | 0.3536166710158189 | 0.44220000381271046 | 0.5602166590591272 | 0.6485499938329061 |
| 32 | 0.18055000031987825 | 0.3109000014762084 | 0.43096666410565376 | 0.5232166647911072 |
| 100 | 0.05973333368698756 | 0.13184999550382295 | 0.21553332979480425 | 0.311366672317187 |
| 316 | 0.002283332869410515 | 0.02449999873836835 | 0.06513333196441333 | 0.13513333598772684 |
| 1000 | -0.007650000353654225 | -0.0019000004976987839 | -8.33341230948766e-05 | -0.002433331683278084 |
### Chart
| Category | 128 | 256 | 512 | 1024 |
|---|---|---|---|---|
| 0 | 0.2536500034232934 | 0.31251666819055873 | 0.3707499938706557 | 0.43993333478768665 |
| 0.1 | 0.5039499985675017 | 0.5788333403567473 | 0.7007000086208185 | 0.805583323041598 |
| 0.32 | 0.4564333346982797 | 0.5508333233495554 | 0.6483333247403303 | 0.7381500055392584 |
| 1 | 0.41170000409086543 | 0.4766000049809615 | 0.6074333327511946 | 0.6835833440224329 |
| 3.2 | 0.41056667144099873 | 0.463500006745259 | 0.5699499982098738 | 0.6607999950647354 |
| 10 | 0.3243500031530857 | 0.4057000018656254 | 0.4604833262662093 | 0.6169500003258387 |
| 32 | 0.2572666642566522 | 0.2708500015238921 | 0.3432333307961623 | 0.46356667081514996 |
| 100 | 0.08234999949733417 | 0.1307000033557415 | 0.19094999755422273 | 0.283050000667572 |
| 316 | 0.022699999312559765 | 0.041450001299381256 | 0.09043333306908607 | 0.16654999802509943 |
| 1000 | 0.004033333311478297 | 0.0030833339939514794 | 0.0021333321928977966 | -0.0027000010013580322 |
### Chart
| Category | 128 | 256 | 512 | 1024 |
|---|---|---|---|---|
| 0 | 0.2536500034232934 | 0.31251666819055873 | 0.3707499938706557 | 0.43993333478768665 |
| 0.1 | 0.449413331101338 | 0.5495000022153059 | 0.6630666740238667 | 0.7802916665871937 |
| 0.32 | 0.38350000356634456 | 0.4978999880452951 | 0.6217333214978377 | 0.706675002972285 |
| 1 | 0.33006666476527846 | 0.42350001012285554 | 0.5288999912639458 | 0.6531750112771988 |
| 3.2 | 0.3491833296914896 | 0.39133334035674733 | 0.5269833294053872 | 0.6040583252906799 |
| 10 | 0.24758333340287209 | 0.36089999849597615 | 0.40284999584158265 | 0.5682583252588908 |
| 32 | 0.20868332559863723 | 0.22033333157499635 | 0.29521666839718824 | 0.41437500218550366 |
| 100 | 0.025516662746667848 | 0.10668333495656651 | 0.14458332583308223 | 0.24894166489442193 |
| 316 | -0.020766672988732655 | 0.021716676652431488 | 0.07666666184862458 | 0.14874165505170825 |
| 1000 | 0.0026833278437455683 | 0.01526666246354578 | 0.01239999383687973 | 0.017775004108746828 |
### Chart
| Category | 128 | 256 | 512 | 1024 | |
|---|---|---|---|---|---|
| 0 | 0.21225000048677126 | 0.2979666627943516 | 0.29428333913286525 | 0.43228333195050556 | None |
| 0.1 | 0.5707166753709316 | 0.6148333288729191 | 0.7592666583756607 | 0.9315000126759208 | None |
| 0.32 | 0.4746833307047685 | 0.5722833462059498 | 0.644433328260978 | 0.8040833373864488 | None |
| 1 | 0.45784999057650566 | 0.5010000007847946 | 0.5989833362400532 | 0.7834000041087463 | None |
| 3.2 | 0.4088500055174033 | 0.404466662555933 | 0.5058999893565972 | 0.6626833279927568 | None |
| 10 | 0.3536166710158189 | 0.4035333332916101 | 0.4920666652421157 | 0.6452833314736681 | None |
| 32 | 0.18055000031987825 | 0.2811166731019815 | 0.3628166603545348 | 0.45054999987284305 | None |
| 100 | 0.05973333368698756 | 0.10481667146086696 | 0.1704500007132689 | 0.27165002127488413 | None |
| 316 | 0.002283332869410515 | -0.0011333264410495585 | 0.03459999834497772 | 0.14193334678808808 | None |
| 1000 | -0.007650000353654225 | 0.01138333665827912 | 0.002383329595128695 | 0.043400012577572866 | None |Hydrocortisone succinate (μg/mL)
Hydrocortisone succinate (μg/mL)
### Chart
| Category | 128 | 256 | 512 | 1024 |
|---|---|---|---|---|
| 0 | 0.1555833319822947 | 0.2279666687051455 | 0.28823333978652954 | 0.361566665271918 |
| 0.01 | 0.1553833211461703 | 0.24929999560117722 | 0.28066666424274445 | 0.3957166746258731 |
| 3.2000000000000001E-2 | 0.1191666598121329 | 0.22863332678874337 | 0.2762333403031031 | 0.40388333549102107 |
| 0.1 | 0.12861666828393978 | 0.23120000710090005 | 0.29616667330265045 | 0.4149500106771783 |
| 0.32 | 0.15016665558020315 | 0.22848333666721982 | 0.2791333297888438 | 0.3996000016729033 |
| 1 | 0.15124999731779137 | 0.2693499947587649 | 0.3121333370606104 | 0.4603666638334588 |
| 3.2 | 0.19048331926266393 | 0.30703333765268326 | 0.34111666679382324 | 0.4529833520452178 |
| 10 | 0.17138333370288253 | 0.28861666967471444 | 0.35626667737960815 | 0.48213335126638374 |
| 32 | 0.10036665946245235 | 0.15638333807388943 | 0.24673333267370862 | 0.4019833380977309 |
| 100 | -0.007283342381318003 | 0.06448333834608397 | 0.1125666672984759 | 0.2834833388527228 |
### Chart
| Category | 128 | 256 | 512 | 1024 |
|---|---|---|---|---|
| 0 | 0.1555833319822947 | 0.2279666687051455 | 0.28823333978652954 | 0.361566665271918 |
| 0.01 | 0.16896666586399078 | 0.263366661965847 | 0.3033499966065089 | 0.3930499975879987 |
| 3.2000000000000001E-2 | 0.18338333318630853 | 0.2588333263993263 | 0.30630000432332355 | 0.4197833314538002 |
| 0.1 | 0.21330000211795172 | 0.2750500018397967 | 0.34363333384195965 | 0.4402666712800662 |
| 0.32 | 0.22066666185855865 | 0.29955000430345535 | 0.3475666642189026 | 0.4563833400607109 |
| 1 | 0.24859999865293503 | 0.30801666527986526 | 0.38028333087762195 | 0.46363332619269687 |
| 3.2 | 0.2622166648507118 | 0.3368166660269101 | 0.4092666705449422 | 0.5256500169634819 |
| 10 | 0.2383833353718122 | 0.31564999371767044 | 0.4013500064611435 | 0.5218500023086866 |
| 32 | 0.1418166682124138 | 0.18201666325330734 | 0.2772666662931442 | 0.39518332729736966 |
| 100 | 0.021066665649414062 | 0.05120000119010607 | 0.11010000358025233 | 0.2376499945918719 |
### Chart
| Category | 128 | 256 | 512 | 1024 |
|---|---|---|---|---|
| 0 | 0.24998333429296812 | 0.2589000016450882 | 0.34326666841904324 | 0.3577750027179718 |
| 0.01 | 0.2741500027477741 | 0.32625000178813934 | 0.36844999343156815 | 0.3890249952673912 |
| 3.2000000000000001E-2 | 0.28291666880249977 | 0.321233332157135 | 0.394849993288517 | 0.45098332564036053 |
| 0.1 | 0.3170333293577035 | 0.32854999601840973 | 0.41305000334978104 | 0.4717666705449422 |
| 0.32 | 0.33034999544421834 | 0.36526666581630707 | 0.4255166674653689 | 0.4995666742324829 |
| 1 | 0.32181666667262715 | 0.4044666687647502 | 0.44993332773447037 | 0.5036333401997884 |
| 3.2 | 0.3570000020166238 | 0.41686666508515674 | 0.46640000492334366 | 0.5375166734059652 |
| 10 | 0.35103332872192067 | 0.341416671872139 | 0.5186833267410597 | 0.5166833301385244 |
| 32 | 0.11353332921862602 | 0.12899999817212424 | 0.25304999699195224 | 0.3071666657924652 |
| 100 | 0.045183333257834114 | 0.0442500039935112 | 0.14278333385785422 | 0.24873333672682443 |
### Chart
| Category | 128 | 256 | 512 | 1024 |
|---|---|---|---|---|
| 0 | 0.24998333429296812 | 0.2589000016450882 | 0.34326666841904324 | 0.3577750027179718 |
| 0.01 | 0.21961333528161048 | 0.296916663646698 | 0.33081665883461636 | 0.363733338812987 |
| 3.2000000000000001E-2 | 0.20998333767056465 | 0.26829999685287476 | 0.3682499900460243 | 0.4195083230733872 |
| 0.1 | 0.23539999003211654 | 0.27545000116030377 | 0.3345166618625323 | 0.44135833779970807 |
| 0.32 | 0.2689666536947092 | 0.2930999994277954 | 0.38254999866088235 | 0.44282500445842743 |
| 1 | 0.24504999692241353 | 0.35966666539510095 | 0.3922999973098437 | 0.4549416651328405 |
| 3.2 | 0.30841666335860884 | 0.366349995136261 | 0.4183833425243696 | 0.4883250047763189 |
| 10 | 0.29419999197125435 | 0.317400003472964 | 0.47231665501991915 | 0.4825749943653743 |
| 32 | 0.0700666569173336 | 0.10926667352517447 | 0.23928332577149075 | 0.28935832281907403 |
| 100 | 0.043833327790101385 | 0.0564333324631055 | 0.15304999550183615 | 0.2692083418369293 |Cell number at Day 14 (absorbance at 560 nm)
Cell number at Day 14 (absorbance at 560 nm)
Quercetin (μM)
Quercetin (μM)
### Chart
| Category | 128 | 256 | 512 | 1024 |
|---|---|---|---|---|
| 0 | 0.14666666959722838 | 0.22161667173107466 | 0.2507166638970375 | 0.3675999939441681 |
| 5.0000000000000001E-3 | 0.16569999977946281 | 0.25056666259964305 | 0.30229999870061874 | 0.41146667301654816 |
| 1.6E-2 | 0.18624999498327574 | 0.2739999902745088 | 0.2909499978025754 | 0.4009833335876465 |
| 0.05 | 0.22310000113745532 | 0.2723166656990846 | 0.31898333380619687 | 0.41190000375111896 |
| 0.16 | 0.2150666651626428 | 0.2808333250383536 | 0.3203333392739296 | 0.38489999373753864 |
| 0.5 | 0.22188333297769228 | 0.28248333061734837 | 0.31955000509818393 | 0.4037666668494542 |
| 1.6 | 0.22873332972327867 | 0.3001999966800213 | 0.32124999910593033 | 0.40416666865348816 |
| 5 | 0.24664999917149544 | 0.2804166687031587 | 0.30871666719516117 | 0.3906333347161611 |
| 15.8 | 0.22693332905570665 | 0.29358333473404247 | 0.30918333182732266 | 0.4212000022331874 |
| 50 | 0.0727166657646497 | 0.20288332924246788 | 0.2253999983270963 | 0.2697666734457016 |
### Chart
| Category | 128 | 256 | 512 | 1024 |
|---|---|---|---|---|
| 0 | 0.14666666959722838 | 0.22161667173107466 | 0.2507166638970375 | 0.3675999939441681 |
| 5.0000000000000001E-3 | 0.15211665506164232 | 0.23649999623497325 | 0.2796166663368543 | 0.4141333500544226 |
| 1.6E-2 | 0.1220333216091001 | 0.24379999066392583 | 0.26088333378235495 | 0.38508333762486735 |
| 0.05 | 0.13841666730344337 | 0.2284666709601879 | 0.27151667326688766 | 0.38658334314823106 |
| 0.16 | 0.1445666588842873 | 0.20976665740211808 | 0.2519000048438708 | 0.32811665534973106 |
| 0.5 | 0.12453333164254862 | 0.24381666009624803 | 0.2514000112811724 | 0.40050000449021617 |
| 1.6 | 0.15699998413523078 | 0.2704166683057944 | 0.25309999535481137 | 0.33150000373522404 |
| 5 | 0.17964999750256577 | 0.2533833446602027 | 0.2636333381136258 | 0.3509166836738582 |
| 15.8 | 0.18548332030574521 | 0.26795000955462456 | 0.27864999820788705 | 0.4280000130335486 |
| 50 | 0.04436665773391764 | 0.21616666639844578 | 0.22786666204531988 | 0.31560001770655255 |
### Chart
| Category | 128 | 256 | 512 | 1024 |
|---|---|---|---|---|
| 0 | 0.223150002459685 | 0.2691500037908554 | 0.3680666660269101 | 0.43974998965859413 |
| 5.0000000000000001E-3 | 0.2541166618466377 | 0.29865000148614246 | 0.37710000326236087 | 0.4730999954044819 |
| 1.6000000000000001E-3 | 0.2679666702946027 | 0.3114333301782608 | 0.40410000334183377 | 0.49641667678952217 |
| 0.05 | 0.26038334021965664 | 0.3158000061909358 | 0.38961666574080783 | 0.47501667216420174 |
| 0.16 | 0.2714333310723305 | 0.31318333248297375 | 0.38590000321467716 | 0.4945666678249836 |
| 0.5 | 0.2788333371281624 | 0.32186666627724964 | 0.39463333040475845 | 0.4542500115931034 |
| 1.6 | 0.27916666120290756 | 0.29809999465942383 | 0.3594500000278155 | 0.45391665771603584 |
| 5 | 0.2446666732430458 | 0.2821999986966451 | 0.3416166678071022 | 0.41624999915560085 |
| 15.8 | 0.2563333387176196 | 0.27203333377838135 | 0.3542833353082339 | 0.42008333280682564 |
| 50 | 0.14878333856662115 | 0.22353333234786987 | 0.28968332956234616 | 0.3042166667679946 |
### Chart
| Category | 128 | 256 | 512 | 1024 |
|---|---|---|---|---|
| 0 | 0.223150002459685 | 0.2691500037908554 | 0.3680666660269101 | 0.43974998965859413 |
| 5.0000000000000001E-3 | 0.19957999438047408 | 0.2693166633447011 | 0.3394666686654091 | 0.44780833895007766 |
| 1.6000000000000001E-3 | 0.19503333916266757 | 0.25849999487400055 | 0.3775000000993411 | 0.46494167422254884 |
| 0.05 | 0.17875000089406967 | 0.26270001133282983 | 0.3110833242535591 | 0.4446083394189676 |
| 0.16 | 0.21004998932282132 | 0.2410166660944621 | 0.3429333344101906 | 0.4378249980509281 |
| 0.5 | 0.20206666737794876 | 0.2770666629076004 | 0.3369999999801318 | 0.40555833652615547 |
| 1.6 | 0.2305833225448926 | 0.24758332471052807 | 0.31143333762884146 | 0.40472498908638954 |
| 5 | 0.1878333364923795 | 0.2581833302974701 | 0.2952499960859617 | 0.38214166338245076 |
| 15.8 | 0.21286666641632718 | 0.2523000091314316 | 0.3405166640877724 | 0.40227498983343446 |
| 50 | 0.14743333309888842 | 0.23571666081746417 | 0.2999499912063281 | 0.32469167187809944 |Astaxanthin (μg/mL)
Astaxanthin (μg/mL)
### Chart: Blank
| Category | 128 | 256 | 512 | 1024 |
|---|---|---|---|---|
| 1 | 0.20773332814375559 | 0.29963333408037823 | 0.3395833373069763 | 0.456333340456088 |
| 2 | 0.22131667286157608 | 0.31370000044504803 | 0.3622666696707408 | 0.45366666341821354 |
| 3 | 0.27195000151793164 | 0.3298333336909612 | 0.3696500013271968 | 0.4722333364188671 |
| 4 | 0.29241666197776794 | 0.3434833288192749 | 0.3870499978462855 | 0.4816500010589759 |
| 5 | 0.2782333344221115 | 0.37070000171661377 | 0.4080166717370351 | 0.5131166788438956 |
| 6 | 0.30508332947889966 | 0.3383000046014786 | 0.40773333112398785 | 0.45960000281532604 |
| 7 | 0.2794666737318039 | 0.3294166624546051 | 0.4077333410580953 | 0.5290000053743521 |
| 8 | 0.27473332981268567 | 0.32666665812333423 | 0.38466666638851166 | 0.49604999149839085 |
| 9 | 0.24918333689371744 | 0.32526665925979614 | 0.37011667092641193 | 0.44953332965572673 |
| 10 | 0.23608333617448807 | 0.28634999692440033 | 0.33711667358875275 | 0.410499996195237 |
### Chart: Blank
| Category | 128 | 256 | 512 | 1024 |
|---|---|---|---|---|
| 1 | 0.2681833282113075 | 0.30516666918992996 | 0.3769499982396762 | 0.5074249990284443 |
| 2 | 0.32271999567747117 | 0.3345000073313713 | 0.41458333283662796 | 0.5327166554828485 |
| 3 | 0.34111665934324265 | 0.3581000044941902 | 0.40355000148216885 | 0.5389000015954176 |
| 4 | 0.3498166675368945 | 0.3582666640480359 | 0.4554833397269249 | 0.5378333317736784 |
| 5 | 0.3295666699608167 | 0.3773333355784416 | 0.41991666704416275 | 0.5641666688024998 |
| 6 | 0.34494999796152115 | 0.3499666725595792 | 0.4345833286643028 | 0.5561166740953922 |
| 7 | 0.3167666668693225 | 0.3556833391388257 | 0.42496666063865024 | 0.5566166676580906 |
| 8 | 0.32501666496197384 | 0.32918333758910495 | 0.4233166699608167 | 0.5415333348015944 |
| 9 | 0.31165000051259995 | 0.32489999383687973 | 0.39071666946013767 | 0.5252333420018355 |
| 10 | 0.26953333367904025 | 0.29298334072033566 | 0.36668333659569424 | 0.48694999391833943 |Inoculated cell
number / well
Inoculated cell
number / well
Row number of 96-microwell plate
Row number of 96-microwell plate
*
*
p <0.05
*
*
*
*
*
*
*
*
*
*
*
*
*
*
*
*
*
*
*
*
*
*
*
*
*
*
*
*
*
*
*
*
*
*
N.S.
*
*
*
*
*
*
*
*
*
*
*
*
*
*
*
*
*
*
*
*
*
*
*
*
*
*
*
*
*
*
*
*
*
*
N.S.
| Inoculated | Hydrocortizone succinate | | | Quercetin | | | Astaxanthin | |
| --- | --- | --- | --- | --- | --- | --- | --- | --- |
| cell number/well | Exp. 1 | Exp. 2 | | Exp. 1 | Exp. 2 | | Exp. 1 | Exp. 2 |
| 128 | 162 | 77 | | 22 | 23 | | 26 | 3 |
| 256 | 106 | 76 | | 35 | 42 | | 22 | 3 |
| 512 | 158 | 79 | | 24 | 38 | | 12 | 0 |
| 1024 | 115 | 77 | | 33 | 36 | | 16 | 6 |

## Slide 3
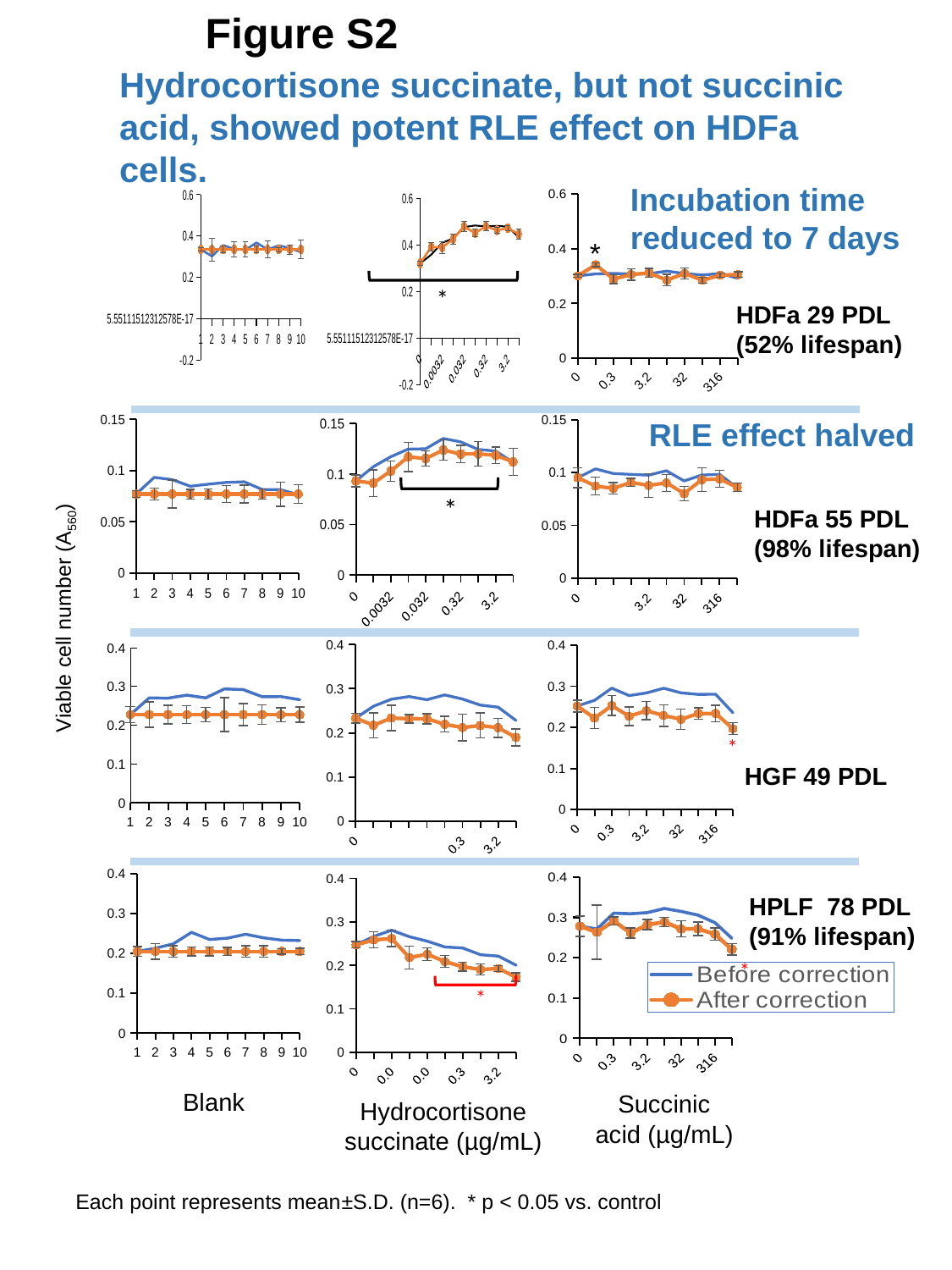

Figure S2
Hydrocortisone succinate, but not succinic acid, showed potent RLE effect on HDFa cells.
Incubation time reduced to 7 days
### Chart
| Category | Before correction | After correction |
|---|---|---|
| 1 | 0.33521665756901103 | 0.33521665756901103 |
| 2 | 0.30176666751503944 | 0.33521665756901103 |
| 3 | 0.3555833262701829 | 0.33521665756901103 |
| 4 | 0.33701667313774425 | 0.33521665756901103 |
| 5 | 0.3326333276927471 | 0.33521665756901103 |
| 6 | 0.36779999857147533 | 0.33521665756901103 |
| 7 | 0.3345666639506817 | 0.33521665756901103 |
| 8 | 0.3543166679640611 | 0.33521665756901103 |
| 9 | 0.34121666476130486 | 0.33521665756901103 |
| 10 | 0.32163333520293236 | 0.33521665756901103 |
### Chart
| Category | Before correction | After correction |
|---|---|---|
| 0 | 0.3008500002324581 | 0.3008500002324581 |
| 0.1 | 0.3081333401302497 | 0.34158333018422127 |
| 0.32 | 0.3096500001847744 | 0.2892833314836025 |
| 1 | 0.307583333303531 | 0.3057833177347978 |
| 3.2 | 0.3087333304186662 | 0.3113166602949301 |
| 10 | 0.31815000002582866 | 0.28556665902336437 |
| 32 | 0.30898332720001537 | 0.3096333208183447 |
| 100 | 0.30385000134507817 | 0.2847499909500281 |
| 316 | 0.3093500000735124 | 0.30334999288121856 |
| 1000 | 0.29195000355442363 | 0.3055333259205023 |
### Chart
| Category | Before correction | After correction |
|---|---|---|
| 0 | 0.3214999983708064 | 0.3214999983708064 |
| 1E-3 | 0.35846665998299915 | 0.3919166500369708 |
| 3.2000000000000002E-3 | 0.40958333015441895 | 0.38921666145324707 |
| 0.01 | 0.42803333202997845 | 0.42623331646124524 |
| 3.2000000000000001E-2 | 0.4772166758775711 | 0.479800005753835 |
| 0.1 | 0.4840499957402547 | 0.4514666547377904 |
| 0.32 | 0.47989999254544574 | 0.48054998616377514 |
| 1 | 0.4826500018437703 | 0.4635499914487203 |
| 3.2 | 0.47868333260218304 | 0.4726833254098892 |
| 10 | 0.43301666776339215 | 0.4465999901294708 |*
*
HDFa 29 PDL
(52% lifespan)
### Chart
| Category | Before correction | After correction |
|---|---|---|
| 1 | 0.0770333285133044 | 0.0770333285133044 |
| 2 | 0.0932833304007848 | 0.07703332851330441 |
| 3 | 0.09109999736150105 | 0.07703332851330441 |
| 4 | 0.08464999745289485 | 0.0770333285133044 |
| 5 | 0.08670000235239665 | 0.0770333285133044 |
| 6 | 0.08844999969005585 | 0.0770333285133044 |
| 7 | 0.08898333211739858 | 0.07703332851330441 |
| 8 | 0.08138333261013031 | 0.0770333285133044 |
| 9 | 0.08130000034968059 | 0.0770333285133044 |
| 10 | 0.07654999817411105 | 0.0770333285133044 |
### Chart
| Category | Before correction | After correction |
|---|---|---|
| 0 | 0.09516666705409686 | 0.09516666705409686 |
| 0.1 | 0.10346666847666104 | 0.08721666658918065 |
| 0.32 | 0.099116666863362 | 0.08504999801516534 |
| 1 | 0.09821666652957599 | 0.09059999758998553 |
| 3.2 | 0.09750000263253848 | 0.08783332879344623 |
| 10 | 0.10171666617194812 | 0.09029999499519666 |
| 32 | 0.09206666673223178 | 0.0801166631281376 |
| 100 | 0.09763333077232043 | 0.09328332667549451 |
| 316 | 0.09820000206430753 | 0.09393333022793134 |
| 1000 | 0.08543333535393079 | 0.08591666569312413 |
### Chart
| Category | Before correction | After correction |
|---|---|---|
| 0 | 0.09305000429352124 | 0.09305000429352124 |
| 1E-3 | 0.10713333760698636 | 0.09088333571950596 |
| 3.2000000000000002E-3 | 0.11694999660054843 | 0.10288332775235177 |
| 0.01 | 0.12448333327968915 | 0.1168666643400987 |
| 3.2000000000000001E-2 | 0.12483333423733711 | 0.11516666039824486 |
| 0.1 | 0.13496666525801024 | 0.12354999408125877 |
| 0.32 | 0.13163333013653755 | 0.11968332653244339 |
| 1 | 0.12414999927083652 | 0.11979999517401059 |
| 3.2 | 0.1226000003516674 | 0.11833332851529121 |
| 10 | 0.11141666894157727 | 0.11189999928077062 |
*
HDFa 55 PDL
(98% lifespan)
Viable cell number (A560)
### Chart
| Category | Before correction | After correction |
|---|---|---|
| 1 | 0.22749999538064003 | 0.22749999538064003 |
| 2 | 0.2705999972919623 | 0.22749999538064 |
| 3 | 0.27015000209212303 | 0.22749999538064003 |
| 4 | 0.2777833354969819 | 0.22749999538064006 |
| 5 | 0.27078333124518394 | 0.22749999538064003 |
| 6 | 0.293799997617801 | 0.22749999538064006 |
| 7 | 0.2920333308478196 | 0.22749999538064006 |
| 8 | 0.2738500001529853 | 0.22749999538064 |
| 9 | 0.274150005231301 | 0.22749999538064 |
| 10 | 0.26606667165954906 | 0.22749999538064006 |
### Chart
| Category | Before correction | After correction |
|---|---|---|
| 0 | 0.2332000012199084 | 0.2332000012199084 |
| 1E-3 | 0.26011667400598526 | 0.21701667209466294 |
| 3.2000000000000002E-3 | 0.2760499988993009 | 0.2333999921878179 |
| 0.01 | 0.28220000118017197 | 0.2319166610638301 |
| 3.2000000000000001E-2 | 0.2751000051697095 | 0.23181666930516562 |
| 0.1 | 0.28575000415245694 | 0.21945000191529596 |
| 0.32 | 0.27638333787520725 | 0.21185000240802768 |
| 1 | 0.2628000055750211 | 0.21645000080267585 |
| 3.2 | 0.258183332780997 | 0.21153332293033597 |
| 10 | 0.22816667209068933 | 0.18959999581178033 |
### Chart
| Category | Before correction | After correction |
|---|---|---|
| 0 | 0.25193333129088086 | 0.25193333129088086 |
| 0.1 | 0.2656833330790202 | 0.22258333116769788 |
| 0.32 | 0.29545000195503235 | 0.25279999524354935 |
| 1 | 0.2773333340883255 | 0.22704999397198364 |
| 3.2 | 0.2837666670481364 | 0.24048333118359247 |
| 10 | 0.2952333390712738 | 0.22893333683411285 |
| 32 | 0.28396667043368023 | 0.21943333496650064 |
| 100 | 0.2801999896764755 | 0.23384998490413025 |
| 316 | 0.2805166741212209 | 0.2338666642705599 |
| 1000 | 0.23541667064030966 | 0.19684999436140063 |*
HGF 49 PDL
### Chart
| Category | Before correction | After correction |
|---|---|---|
| 1 | 0.20478333036104837 | 0.20478333036104837 |
| 2 | 0.2128833308815956 | 0.20478333036104837 |
| 3 | 0.22391666968663534 | 0.20478333036104834 |
| 4 | 0.25283333162466687 | 0.20478333036104834 |
| 5 | 0.23480000098546347 | 0.20478333036104834 |
| 6 | 0.23826666673024496 | 0.20478333036104834 |
| 7 | 0.24798333644866943 | 0.20478333036104837 |
| 8 | 0.23891666531562805 | 0.20478333036104837 |
| 9 | 0.23316665987173715 | 0.20478333036104837 |
| 10 | 0.23223333060741425 | 0.20478333036104837 |
### Chart
| Category | Before correction | After correction |
|---|---|---|
| 0 | 0.2783333286643028 | 0.2783333286643028 |
| 0.1 | 0.2709833284219106 | 0.2628833279013633 |
| 0.32 | 0.31048332899808884 | 0.29134998967250186 |
| 1 | 0.30896666397651035 | 0.2609166627128919 |
| 3.2 | 0.31181666006644565 | 0.28179998944203055 |
| 10 | 0.32189999769131344 | 0.2884166613221168 |
| 32 | 0.3146333321928978 | 0.27143332610527665 |
| 100 | 0.3055666660269101 | 0.2714333310723304 |
| 316 | 0.2867833251754443 | 0.2583999956647555 |
| 1000 | 0.24806666622559229 | 0.22061666597922644 |
### Chart
| Category | Before correction | After correction |
|---|---|---|
| 0 | 0.24731666470567384 | 0.24731666470567384 |
| 1E-3 | 0.2665333313246568 | 0.2584333308041096 |
| 3.2000000000000002E-3 | 0.28076666221022606 | 0.2616333228846391 |
| 0.01 | 0.2659166616698106 | 0.2178666604061921 |
| 3.2000000000000001E-2 | 0.25564999505877495 | 0.22563332443435982 |
| 0.1 | 0.24223333224654198 | 0.20874999587734536 |
| 0.32 | 0.23985000078876814 | 0.19664999470114708 |
| 1 | 0.22443333392341933 | 0.19029999896883965 |
| 3.2 | 0.22123332942525545 | 0.19284999991456667 |
| 10 | 0.20014999931057295 | 0.17269999906420708 |HPLF 78 PDL
(91% lifespan)
*
*
Blank
Succinic acid (µg/mL)
Hydrocortisone succinate (µg/mL)
RLE effect halved
Each point represents mean±S.D. (n=6). * p < 0.05 vs. control

## Slide 4
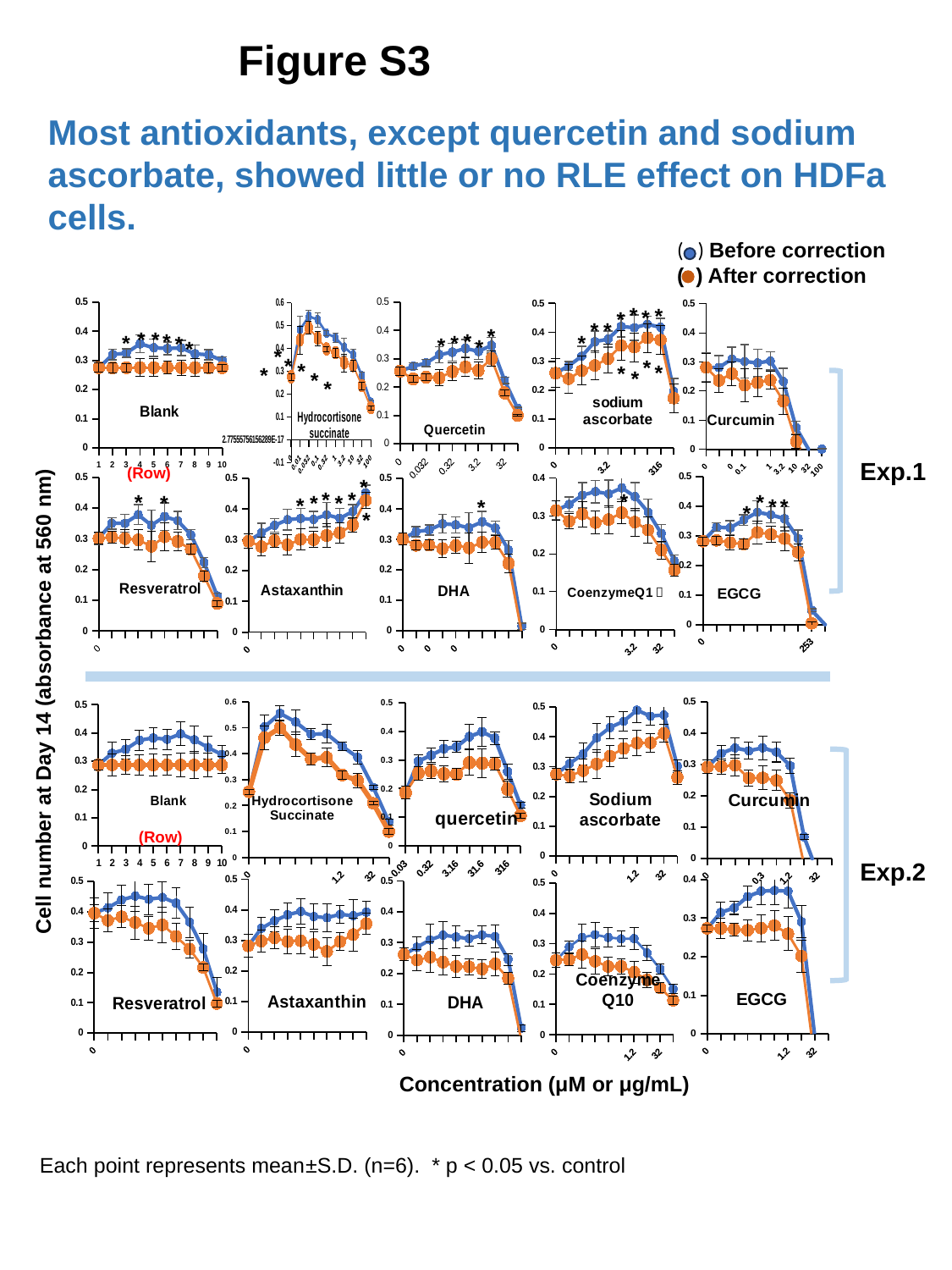

Figure S3
Most antioxidants, except quercetin and sodium ascorbate, showed little or no RLE effect on HDFa cells.
( ) Before correction
( ) After correction
### Chart: Quercetin
| Category | | |
|---|---|---|
| 0 | 0.25591666996479034 | 0.25591666996479034 |
| 0.01 | 0.2729833374420802 | 0.22823333740234417 |
| 3.2000000000000001E-2 | 0.2846333384513855 | 0.23468333482742354 |
| 0.1 | 0.3139999955892563 | 0.23226666450500533 |
| 0.32 | 0.32276666661103565 | 0.25466667115688374 |
| 1 | 0.33631666501363117 | 0.2705499976873403 |
| 3.2 | 0.325766662756602 | 0.2582000046968464 |
| 10 | 0.34693333009878796 | 0.2995999952157343 |
| 32 | 0.2232499967018763 | 0.17914999524752342 |
| 100 | 0.12445000062386195 | 0.10010000814994224 |
### Chart: sodium ascorbate
| Category | | |
|---|---|---|
| 0 | 0.2586499924461047 | 0.2586499924461047 |
| 0.1 | 0.2837500000993411 | 0.23900000005960506 |
| 0.32 | 0.31734999269247055 | 0.2673999890685086 |
| 1 | 0.3674166624744733 | 0.28568333139022234 |
| 3.2 | 0.37656666586796445 | 0.3084666704138125 |
| 10 | 0.42009999603033066 | 0.3543333287040397 |
| 31.6 | 0.41591665893793106 | 0.3483500008781755 |
| 100 | 0.4279166633884112 | 0.38058332850535753 |
| 316 | 0.41734999666611355 | 0.37324999521176067 |
| 1000 | 0.19651666780312857 | 0.17216667532920885 |
### Chart: Curcumin
| Category | | |
|---|---|---|
| 0 | 0.28108333175381023 | 0.28108333175381023 |
| 0.01 | 0.28138333186507225 | 0.23663333182533622 |
| 3.2000000000000001E-2 | 0.3100166631241639 | 0.26006665950020197 |
| 0.1 | 0.301983330398798 | 0.22024999931454703 |
| 0.32 | 0.29668334250648815 | 0.2285833470523362 |
| 1 | 0.3035666656990846 | 0.23779999837279367 |
| 3.2 | 0.232849999020497 | 0.16528334096074146 |
| 10 | 0.0741833324233691 | 0.026849997540315456 |
| 32 | -0.002166666090488434 | -0.04626666754484132 |
| 100 | 0.0006166671713193258 | -0.0237333253026004 |
### Chart: Hydrocortisone succinate
| Category | | |
|---|---|---|
| 0 | 0.2763499977687995 | 0.2763499977687995 |
| 0.01 | 0.4817499928176403 | 0.4369999927779043 |
| 3.2000000000000001E-2 | 0.5406833353141943 | 0.4907333316902324 |
| 0.1 | 0.5260166687270006 | 0.4442833376427496 |
| 0.32 | 0.4662333292265733 | 0.3981333337724213 |
| 1 | 0.4476666661600272 | 0.3818999988337362 |
| 3.2 | 0.40540000920494396 | 0.3378333511451885 |
| 10 | 0.3726499987145265 | 0.3253166638314729 |
| 32 | 0.2809499961634477 | 0.23684999470909482 |
| 100 | 0.16433333232998848 | 0.13998333985606878 |
### Chart: Blank
| Category | | |
|---|---|---|
| 1 | 0.27541666850447655 | 0.27541666850447655 |
| 2 | 0.320166668544213 | 0.27541666850447694 |
| 3 | 0.32536667212843895 | 0.275416668504477 |
| 4 | 0.35714999958872795 | 0.275416668504477 |
| 5 | 0.34351666395862895 | 0.27541666850447705 |
| 6 | 0.34118333583076793 | 0.27541666850447705 |
| 7 | 0.3429833265642325 | 0.27541666850447694 |
| 8 | 0.3227500033875306 | 0.27541666850447705 |
| 9 | 0.3195166699588299 | 0.275416668504477 |
| 10 | 0.2997666609783967 | 0.27541666850447705 |
Exp.1
(Row)
### Chart: EGCG
| Category | | |
|---|---|---|
| 0 | 0.28193333620826405 | 0.28193333620826405 |
| 0.08 | 0.3295000034073989 | 0.2847500033676628 |
| 0.25 | 0.32706667358676594 | 0.277116669962804 |
| 0.8 | 0.35366667186220485 | 0.2719333407779539 |
| 2.5299999999999998 | 0.38003333285450935 | 0.3119333374003574 |
| 8 | 0.3710166700184345 | 0.3052500026921436 |
| 25.3 | 0.3580166685084502 | 0.2904500104486946 |
| 80 | 0.29151667033632594 | 0.24418333545327234 |
| 253 | 0.04851666713754336 | 0.004416665683190472 |
| 800 | -0.0003666648020346959 | -0.02471665727595442 |
### Chart: Resveratrol
| Category | | |
|---|---|---|
| 0 | 0.30116666729251546 | 0.30116666729251546 |
| 4.0000000000000001E-3 | 0.3504499979317188 | 0.3056999978919828 |
| 1.2999999999999999E-2 | 0.3504499979317188 | 0.30049999430775687 |
| 0.04 | 0.3782999999821186 | 0.29656666889786765 |
| 0.126 | 0.3438500054180622 | 0.27575000996391025 |
| 0.4 | 0.37220000599821407 | 0.3064333386719232 |
| 1.26 | 0.35900000110268593 | 0.2914333430429304 |
| 4 | 0.31266666824618977 | 0.26533333336313614 |
| 12.6 | 0.22313333426912627 | 0.17903333281477338 |
| 40 | 0.1139333335061868 | 0.08958334103226707 |
### Chart: Astaxanthin
| Category | | |
|---|---|---|
| 0 | 0.29598332817355794 | 0.29598332817355794 |
| 4.0000000000000001E-3 | 0.32279999926686287 | 0.27804999922712686 |
| 1.2999999999999999E-2 | 0.3473166711628437 | 0.29736666753888175 |
| 0.04 | 0.36549999689062435 | 0.2837666658063734 |
| 0.126 | 0.36971666291356087 | 0.3016166674594089 |
| 0.4 | 0.36668333287040394 | 0.300916665544113 |
| 1.26 | 0.3819166657825311 | 0.31435000772277555 |
| 4 | 0.3701666680475076 | 0.322833333164454 |
| 12.6 | 0.39213333403070766 | 0.34803333257635477 |
| 40 | 0.4528000093996525 | 0.42845001692573276 |
### Chart: CoenzymeQ1０
| Category | | |
|---|---|---|
| 0 | 0.31505000218749046 | 0.31505000218749046 |
| 0.01 | 0.3310999982059002 | 0.2863499981661642 |
| 3.2000000000000001E-2 | 0.3552666666607062 | 0.30531666303674426 |
| 0.1 | 0.3647499990959962 | 0.28301666801174524 |
| 0.32 | 0.35883333906531334 | 0.2907333436111614 |
| 1 | 0.37468333666523296 | 0.3089166693389421 |
| 3.2 | 0.3515833330651124 | 0.2840166750053569 |
| 10 | 0.3105500054856141 | 0.26321667060256054 |
| 32 | 0.2543833317855994 | 0.2102833303312465 |
| 100 | 0.18143333370486894 | 0.15708334123094925 |
### Chart: DHA
| Category | | |
|---|---|---|
| 0 | 0.30194999650120735 | 0.30194999650120735 |
| 4.0000000000000001E-3 | 0.3257666664818923 | 0.2810166664421562 |
| 1.2999999999999999E-2 | 0.3313666669030984 | 0.28141666327913645 |
| 0.04 | 0.3516166644791762 | 0.26988333339492526 |
| 0.126 | 0.3483999955157439 | 0.28030000006159195 |
| 0.4 | 0.33833333229025203 | 0.27256666496396115 |
| 1.26 | 0.3581499941647053 | 0.29058333610494974 |
| 4 | 0.33750000471870106 | 0.29016666983564743 |
| 12.6 | 0.2655999946097533 | 0.2214999931554004 |
| 40 | 0.014899998903274536 | -0.009449993570645188 |Cell number at Day 14 (absorbance at 560 nm)
### Chart: Curcumin
| Category | | |
|---|---|---|
| 0 | 0.2927166670560837 | 0.2927166670560837 |
| 0.01 | 0.33605000376701355 | 0.2944500048955279 |
| 3.2000000000000001E-2 | 0.3536333342393239 | 0.2979166706403094 |
| 0.1 | 0.34441666801770526 | 0.2569333364566165 |
| 0.32 | 0.35376666486263275 | 0.2580499947071073 |
| 1 | 0.3404500037431717 | 0.24955000976721423 |
| 1.2 | 0.29635000725587207 | 0.18620001773039474 |
| 10 | 0.06958333402872086 | -0.019433327019214852 |
| 32 | -0.04808333267768224 | -0.10975000510613149 |
| 100 | -0.045466666420300804 | -0.0827666670084002 |
### Chart: Hydrocortisone Succinate
| Category | | |
|---|---|---|
| 0 | 0.25283333162466687 | 0.25283333162466687 |
| 0.01 | 0.5030666639407476 | 0.46146666506926187 |
| 3.2000000000000001E-2 | 0.5551333377758662 | 0.49941667417685176 |
| 0.1 | 0.5221666743357977 | 0.43468334277470894 |
| 0.32 | 0.4747999956210454 | 0.37908332546552 |
| 1 | 0.47661666572093964 | 0.3857166717449822 |
| 1.2 | 0.4278166840473811 | 0.3176666945219037 |
| 10 | 0.385616660118103 | 0.2965999990701673 |
| 32 | 0.26988333463668823 | 0.208216662208239 |
| 100 | 0.13681666304667792 | 0.09951666245857849 |
### Chart: quercetin
| Category | | |
|---|---|---|
| 0.03 | 0.18541666120290756 | 0.18541666120290756 |
| 0.1 | 0.29508332659800846 | 0.2534833277265228 |
| 0.32 | 0.316300002237161 | 0.2605833386381465 |
| 1 | 0.33886666347583133 | 0.25138333191474255 |
| 3.16 | 0.34601666778326035 | 0.2502999976277349 |
| 10 | 0.3811999981602033 | 0.29030000418424584 |
| 31.6 | 0.39846666405598324 | 0.28831667453050586 |
| 100 | 0.3756166622042656 | 0.2866000011563299 |
| 316 | 0.2593499993284543 | 0.1976833269000051 |
| 1000 | 0.14216666420300803 | 0.1048666636149086 |
### Chart: Blank
| Category | | |
|---|---|---|
| 1 | 0.28696667154630023 | 0.28696667154630023 |
| 2 | 0.32856667041778564 | 0.2869666715463 |
| 3 | 0.3426833351453145 | 0.2869666715463 |
| 4 | 0.3744500031073888 | 0.2869666715463 |
| 5 | 0.38268334170182544 | 0.2869666715463 |
| 6 | 0.3778666655222575 | 0.2869666715463 |
| 7 | 0.39711666107177734 | 0.2869666715463 |
| 8 | 0.3759833325942357 | 0.2869666715463 |
| 9 | 0.34863334397474927 | 0.2869666715463 |
| 10 | 0.3242666721343994 | 0.2869666715463 |
### Chart: Sodium ascorbate
| Category | | |
|---|---|---|
| 0 | 0.27498332659403485 | 0.27498332659403485 |
| 0.01 | 0.3106333315372467 | 0.26903333266576107 |
| 3.2000000000000001E-2 | 0.34263332684834796 | 0.28691666324933346 |
| 0.1 | 0.3967333386341731 | 0.3092500070730843 |
| 0.32 | 0.43139999608198804 | 0.3356833259264626 |
| 1 | 0.4522499938805898 | 0.36134999990463235 |
| 1.2 | 0.48963333666324615 | 0.3794833471377688 |
| 10 | 0.4694499919811885 | 0.38043333093325277 |
| 32 | 0.47361665467421216 | 0.4119499822457629 |
| 100 | 0.3016333281993866 | 0.2643333276112872 |
(Row)
Exp.2
### Chart: Astaxanthin
| Category | | |
|---|---|---|
| 0 | 0.2824333384633064 | 0.2824333384633064 |
| 4.0000000000000001E-3 | 0.33951666702826816 | 0.2979166681567825 |
| 1.2999999999999999E-2 | 0.3639666810631752 | 0.3082500174641607 |
| 0.04 | 0.38370000571012497 | 0.2962166741490362 |
| 0.126 | 0.3948000098268191 | 0.2990833396712937 |
| 0.4 | 0.378050001959006 | 0.2871500079830485 |
| 1.26 | 0.3741166765491168 | 0.2639666870236394 |
| 4 | 0.3853333368897438 | 0.2963166758418081 |
| 12.6 | 0.380683330198129 | 0.3190166577696798 |
| 40 | 0.392283337811629 | 0.3549833372235296 |
### Chart:  Resveratrol
| Category | | |
|---|---|---|
| 0 | 0.3956333336730798 | 0.3956333336730798 |
| 4.0000000000000001E-3 | 0.41341666256388027 | 0.3718166636923947 |
| 1.2999999999999999E-2 | 0.43923334156473476 | 0.38351667796572025 |
| 0.04 | 0.4522833364705245 | 0.3648000049094357 |
| 0.126 | 0.44156667465964955 | 0.3458500045041241 |
| 0.4 | 0.44788333649436635 | 0.35698334251840885 |
| 1.26 | 0.42900000388423604 | 0.31885001435875865 |
| 4 | 0.36603333428502083 | 0.2770166732370851 |
| 12.6 | 0.277883334706227 | 0.21621666227777778 |
| 40 | 0.13470000152786574 | 0.09740000093976632 |
### Chart: EGCG
| Category | | |
|---|---|---|
| 0 | 0.27356665829817456 | 0.27356665829817456 |
| 0.01 | 0.31476666529973346 | 0.27316666642824783 |
| 3.2000000000000001E-2 | 0.32661665976047516 | 0.27089999616146065 |
| 0.1 | 0.35606666405995685 | 0.26858333249886807 |
| 0.32 | 0.36984999974568683 | 0.2741333295901614 |
| 1 | 0.3717166632413864 | 0.280816669265429 |
| 1.2 | 0.36996667087078094 | 0.2598166813453036 |
| 10 | 0.2906999985376994 | 0.20168333748976366 |
| 32 | -0.010116670280694962 | -0.07178334270914422 |
| 100 | -0.06245000349978606 | -0.09975000408788547 |
### Chart: Coenzyme Q10
| Category | | |
|---|---|---|
| 0 | 0.24555000166098276 | 0.24555000166098276 |
| 0.01 | 0.2894333302974701 | 0.24783333142598443 |
| 3.2000000000000001E-2 | 0.32003333667914075 | 0.26431667308012624 |
| 0.1 | 0.3294999897480011 | 0.24201665818691231 |
| 0.32 | 0.3205000013113022 | 0.22478333115577676 |
| 1 | 0.3158666640520096 | 0.22496667007605212 |
| 1.2 | 0.31643333037694293 | 0.2062833408514656 |
| 10 | 0.2694833328326543 | 0.18046667178471862 |
| 32 | 0.21583333611488342 | 0.15416666368643417 |
| 100 | 0.1504999945561091 | 0.11319999396800969 |
### Chart: DHA
| Category | | |
|---|---|---|
| 0 | 0.26213333507378894 | 0.26213333507378894 |
| 4.0000000000000001E-3 | 0.2852666676044464 | 0.24366666873296075 |
| 1.2999999999999999E-2 | 0.3090166747570038 | 0.2533000111579893 |
| 0.04 | 0.32438333829243976 | 0.236900006731351 |
| 0.126 | 0.3188166618347168 | 0.22309999167919137 |
| 0.4 | 0.31293333570162457 | 0.2220333417256671 |
| 1.26 | 0.32473333179950714 | 0.21458334227402978 |
| 4 | 0.32034999628861743 | 0.23133333524068175 |
| 12.6 | 0.2458666612704595 | 0.18419998884201025 |
| 40 | 0.02341666693488757 | -0.013883333653211835 |Concentration (μM or μg/mL)
*
*
*
*
*
*
*
*
*
*
*
*
*
*
*
*
*
*
*
*
*
*
*
*
*
*
*
*
*
*
*
*
*
*
*
*
*
*
*
*
*
*
*
Each point represents mean±S.D. (n=6). * p < 0.05 vs. control

## Slide 5
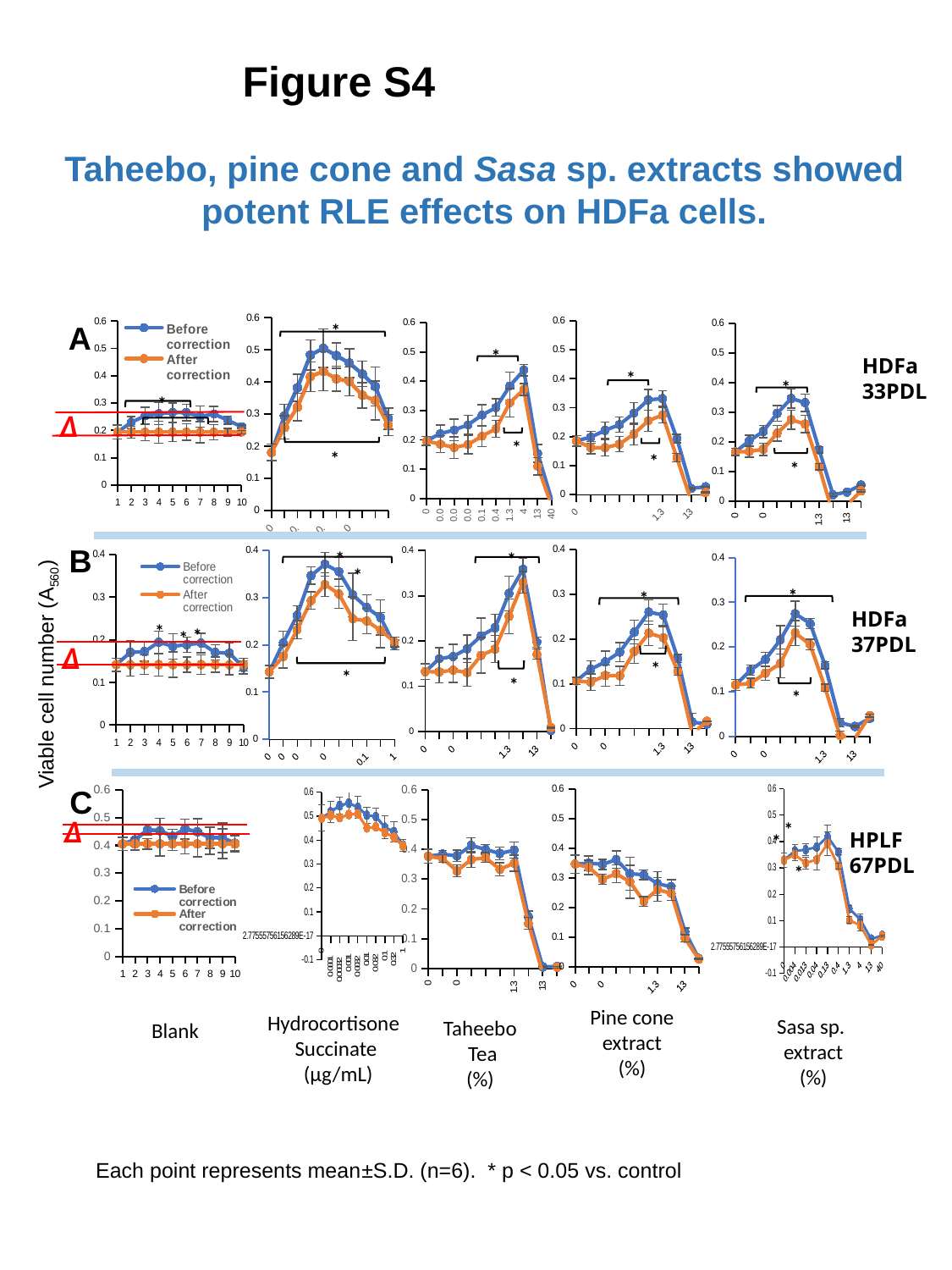

Figure S4
Taheebo, pine cone and Sasa sp. extracts showed potent RLE effects on HDFa cells.
### Chart
| Category | Before correction | After correction |
|---|---|---|
| 0 | 0.1857499989370505 | 0.1857499989370505 |
| 4.0000000000000001E-3 | 0.19805000349879265 | 0.1623500051597754 |
| 1.2999999999999999E-2 | 0.2218333346148332 | 0.16189999754230183 |
| 0.04 | 0.2414833369354407 | 0.17403334006667137 |
| 0.13 | 0.28064999853571254 | 0.20901666457454363 |
| 0.4 | 0.32678332800666493 | 0.25463332608342176 |
| 1.3 | 0.33166666701436043 | 0.2744000044961771 |
| 4 | 0.193466667085886 | 0.12721667066216466 |
| 13 | 0.02160000056028366 | -0.021949999034404755 |
| 40 | 0.02736666922767957 | 0.007616670181353887 |
### Chart
| Category | Before correction | After correction |
|---|---|---|
| 0 | 0.18091666450103125 | 0.18091666450103125 |
| 1E-4 | 0.29453333218892414 | 0.2588333338499069 |
| 3.2000000000000003E-4 | 0.38235000272591907 | 0.3224166656533878 |
| 1E-3 | 0.4844000041484833 | 0.41695000727971404 |
| 3.2000000000000002E-3 | 0.5051666647195816 | 0.43353333075841277 |
| 0.01 | 0.4825166662534078 | 0.41036666433016467 |
| 3.2000000000000001E-2 | 0.4592999964952469 | 0.4020333339770636 |
| 0.1 | 0.42508332928021747 | 0.35883333285649616 |
| 0.32 | 0.38625000417232513 | 0.3427000045776367 |
| 1 | 0.28598332901795703 | 0.26623332997163135 |
### Chart
| Category | Before correction | After correction |
|---|---|---|
| 0 | 0.16531666864951453 | 0.16531666864951453 |
| 4.0000000000000001E-3 | 0.20261666427055994 | 0.16691666593154272 |
| 1.2999999999999999E-2 | 0.234483336408933 | 0.1745499993364016 |
| 0.04 | 0.29563333094120026 | 0.22818333407243094 |
| 0.13 | 0.3455333411693573 | 0.2739000072081884 |
| 0.4 | 0.33156666656335193 | 0.25941666464010876 |
| 1.3 | 0.17266666640837988 | 0.1154000038901965 |
| 4 | 0.020850001523892086 | -0.04539999489982926 |
| 13 | 0.03006666526198387 | -0.013483334332704544 |
| 40 | 0.0536833330988884 | 0.033933334052562714 |*
### Chart
| Category | Before correction | After correction |
|---|---|---|
| 1 | 0.1939499999086062 | 0.1939499999086062 |
| 2 | 0.22964999824762344 | 0.1939499999086062 |
| 3 | 0.2538833369811376 | 0.1939499999086062 |
| 4 | 0.2613999967773755 | 0.1939499999086062 |
| 5 | 0.2655833338697751 | 0.1939499999086062 |
| 6 | 0.2661000018318494 | 0.1939499999086062 |
| 7 | 0.2512166624267896 | 0.1939499999086062 |
| 8 | 0.26019999633232754 | 0.19394999990860617 |
| 9 | 0.23749999950329462 | 0.1939499999086062 |
| 10 | 0.21369999895493189 | 0.1939499999086062 |
### Chart
| Category | Before correction | After correction |
|---|---|---|
| 0 | 0.19608333085974058 | 0.19608333085974058 |
| 4.0000000000000001E-3 | 0.2209833301603794 | 0.18528333182136217 |
| 1.2999999999999999E-2 | 0.23341666534543037 | 0.173483328272899 |
| 0.04 | 0.25140000010530156 | 0.1839500032365322 |
| 0.13 | 0.28433332964777946 | 0.21269999568661055 |
| 0.4 | 0.3098833275338014 | 0.23773332561055818 |
| 1.3 | 0.38366666560371715 | 0.32640000308553385 |
| 4 | 0.4376833302279313 | 0.37143333380421 |
| 13 | 0.1532999984920025 | 0.10974999889731407 |
| 40 | -0.00406666782995065 | -0.023816666876276333 |*
HDFa 33PDL
*
*
*
Δ
*
*
*
*
### Chart
| Category | Before correction | After correction |
|---|---|---|
| 0 | 0.142683328439792 | 0.142683328439792 |
| 1E-4 | 0.2039166676501433 | 0.17484999820590016 |
| 3.2000000000000003E-4 | 0.2624833323061466 | 0.23189999535679817 |
| 1E-3 | 0.34621666247646016 | 0.29344999417662615 |
| 3.2000000000000002E-3 | 0.3703999953965346 | 0.3273833307127158 |
| 0.01 | 0.35473332678278285 | 0.3075833283364772 |
| 3.2000000000000001E-2 | 0.3055499978363514 | 0.25513332709670067 |
| 0.1 | 0.2790333318213622 | 0.25039999807874364 |
| 0.32 | 0.2575666618843873 | 0.23076666022340453 |
| 1 | 0.19968333219488463 | 0.20618333046634993 |*
### Chart
| Category | Before correction | After correction |
|---|---|---|
| 0 | 0.13230000187953314 | 0.13230000187953314 |
| 4.0000000000000001E-3 | 0.16069999585549036 | 0.13163332641124723 |
| 1.2999999999999999E-2 | 0.16593333085378012 | 0.13534999390443167 |
| 0.04 | 0.18290000408887863 | 0.13013333578904465 |
| 0.13 | 0.21129999806483588 | 0.16828333338101706 |
| 0.4 | 0.22914999971787134 | 0.18200000127156576 |
| 1.3 | 0.30513333280881244 | 0.2547166620691617 |
| 4 | 0.35833332935969037 | 0.3296999956170718 |
| 13 | 0.19766666491826376 | 0.17086666325728095 |
| 40 | 0.00199999970694383 | 0.008499997978409132 |*
### Chart
| Category | Before correction | After correction |
|---|---|---|
| 0 | 0.10633333027362823 | 0.10633333027362823 |
| 4.0000000000000001E-3 | 0.132383331656456 | 0.10331666221221285 |
| 1.2999999999999999E-2 | 0.14878333111604056 | 0.1181999941666921 |
| 0.04 | 0.17056666811307272 | 0.11779999981323874 |
| 0.13 | 0.21496666222810745 | 0.17194999754428864 |
| 0.4 | 0.2598833292722702 | 0.2127333308259646 |
| 1.3 | 0.25323332846164703 | 0.2028166577219963 |
| 4 | 0.1563833331068357 | 0.12774999936421713 |
| 13 | 0.014549997945626577 | -0.012250003715356209 |
| 40 | 0.009849998479088148 | 0.016349996750553448 |
### Chart
| Category | Before correction | After correction |
|---|---|---|
| 1 | 0.1417666661242644 | 0.1417666661242644 |
| 2 | 0.17083333556850752 | 0.14176666612426436 |
| 3 | 0.17235000307361284 | 0.1417666661242644 |
| 4 | 0.19453333442409834 | 0.14176666612426436 |
| 5 | 0.1847833308080832 | 0.1417666661242644 |
| 6 | 0.18891666457057 | 0.1417666661242644 |
| 7 | 0.19218333686391512 | 0.1417666661242644 |
| 8 | 0.17039999986688295 | 0.1417666661242644 |
| 9 | 0.16856666778524718 | 0.14176666612426436 |
| 10 | 0.1352666678527991 | 0.1417666661242644 |
### Chart
| Category | Before correction | After correction |
|---|---|---|
| 0 | 0.11526666829983394 | 0.11526666829983394 |
| 4.0000000000000001E-3 | 0.1480166663726171 | 0.11894999692837394 |
| 1.2999999999999999E-2 | 0.17208333313465118 | 0.14149999618530273 |
| 0.04 | 0.2160500039656957 | 0.16328333566586173 |
| 0.13 | 0.27435000240802765 | 0.23133333772420883 |
| 0.4 | 0.2524166653553645 | 0.2052666669090589 |
| 1.3 | 0.1592333341638247 | 0.108816663424174 |
| 4 | 0.030783335367838543 | 0.0021500016252199807 |
| 13 | 0.02230000247557958 | -0.004499999185403207 |
| 40 | 0.0403333343565464 | 0.046833332628011703 |*
*
*
HDFa 37PDL
*
*
*
Δ
*
*
Viable cell number (A560)
*
*
### Chart
| Category | Before correction | After correction |
|---|---|---|
| 0 | 0.34603333473205566 | 0.34603333473205566 |
| 4.0000000000000001E-3 | 0.34940000375111896 | 0.33496666451295215 |
| 1.2999999999999999E-2 | 0.34528333445390064 | 0.2947833289702733 |
| 0.04 | 0.36129999657471973 | 0.3137166599432627 |
| 0.13 | 0.3136499971151352 | 0.2861499985059102 |
| 0.4 | 0.31 | 0.22 |
| 1.3 | 0.28 | 0.26 |
| 4 | 0.2696000039577484 | 0.24745000402132664 |
| 13 | 0.11806666851043701 | 0.09585000077883399 |
| 40 | 0.027466667195161183 | 0.025116654733816763 |
### Chart
| Category | Before correction | After correction |
|---|---|---|
| 0 | 0.32926666860779125 | 0.32926666860779125 |
| 4.0000000000000001E-3 | 0.36445000395178795 | 0.35001666471362114 |
| 1.2999999999999999E-2 | 0.36898333951830864 | 0.3184833340346813 |
| 0.04 | 0.37919999783237773 | 0.3316166612009207 |
| 0.13 | 0.41896667207280797 | 0.391466673463583 |
| 0.4 | 0.36036666855216026 | 0.3070833347737789 |
| 1.3 | 0.14551666751503944 | 0.101833322395881 |
| 4 | 0.10398333643873532 | 0.08183333650231357 |
| 13 | 0.030166668196519215 | 0.007950000464916193 |
| 40 | 0.04376666868726412 | 0.0414166562259197 |
### Chart
| Category | Before correction | After correction |
|---|---|---|
| 0 | 0.3764333340028922 | 0.37643333400289214 |
| 4.0000000000000001E-3 | 0.383266668766737 | 0.3688333295285702 |
| 1.2999999999999999E-2 | 0.37806667014956474 | 0.3275666646659374 |
| 0.04 | 0.41278334086139995 | 0.3652000042299429 |
| 0.13 | 0.3994000020126502 | 0.3719000034034252 |
| 0.4 | 0.38593333090345067 | 0.33264999712506926 |
| 1.3 | 0.39676666880647343 | 0.353083323687315 |
| 4 | 0.17339999849597612 | 0.15124999855955437 |
| 13 | 0.005400001381834348 | -0.016816666349768677 |
| 40 | 0.006783333917458852 | 0.004433321456114432 |
### Chart
| Category | Before correction | After correction |
|---|---|---|
| 1 | 0.40554999684294063 | 0.40554999684294063 |
| 2 | 0.41998333608110744 | 0.40554999684294063 |
| 3 | 0.45605000232656795 | 0.40554999684294063 |
| 4 | 0.45313333347439766 | 0.40554999684294063 |
| 5 | 0.4330499954521656 | 0.40554999684294063 |
| 6 | 0.458833330621322 | 0.40554999684294063 |
| 7 | 0.4492333419620991 | 0.40554999684294063 |
| 8 | 0.4276999967793624 | 0.4055499968429406 |
| 9 | 0.42776666457454365 | 0.4055499968429406 |
| 10 | 0.40790000930428505 | 0.40554999684294063 |
### Chart
| Category | Before correction | After correction |
|---|---|---|
| 0 | 0.49111667027076084 | 0.49111667027076084 |
| 1E-4 | 0.5180166587233543 | 0.5035833194851875 |
| 3.2000000000000003E-4 | 0.5441333378354708 | 0.49363333235184353 |
| 1E-3 | 0.5543999895453453 | 0.5068166529138882 |
| 3.2000000000000002E-3 | 0.5371999964118004 | 0.5096999978025752 |
| 0.01 | 0.5042333329717318 | 0.4509499991933505 |
| 3.2000000000000001E-2 | 0.49828333407640457 | 0.4545999889572461 |
| 0.1 | 0.45385000358025235 | 0.43170000364383054 |
| 0.32 | 0.43415000786383945 | 0.4119333401322364 |
| 1 | 0.3783833359678586 | 0.37603332350651425 |Δ
*
HPLF 67PDL
*
*
Pine cone extract
(%)
Hydrocortisone
Succinate
 (µg/mL)
Sasa sp.
extract
(%)
Taheebo
 Tea
(%)
Blank
A
B
C
Each point represents mean±S.D. (n=6). * p < 0.05 vs. control

## Slide 6
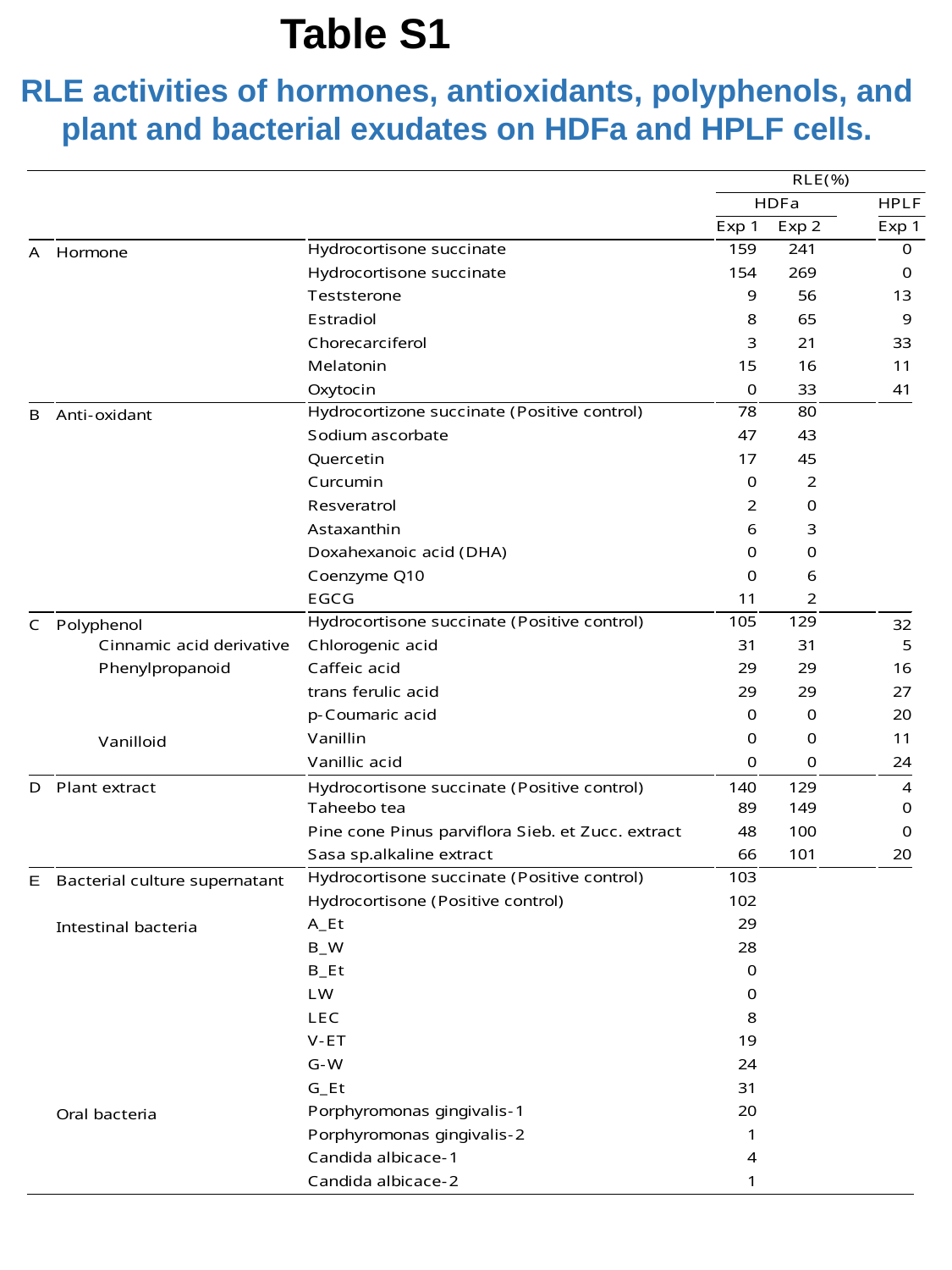

Table S1
RLE activities of hormones, antioxidants, polyphenols, and plant and bacterial exudates on HDFa and HPLF cells.
